# Conservation status assessments of species-rich tropical taxa in the face of data availability limitations: insights from Sulawesi *Begonia*

**DOI:** 10.1038/s41598-024-64319-7

**Published:** 2024-06-18

**Authors:** Daniel C. Thomas, Wisnu H. Ardi, Yu Hong Chong, Philip Thomas, Mark Hughes

**Affiliations:** 1 National Parks Board, Singapore Botanic Gardens, Singapore, Singapore; 2 School of Biological Sciences, Nanyang Technological University, Singapore, Singapore; 3 Research Center for Biosystematics and Evolution, National Research and Innovation Agency, BRIN, Jakarta, Indonesia; 4 Institute of Food and Agricultural Sciences, University of Florida, Gainesville, USA; 5 Royal Botanic Garden, Edinburgh, Scotland, UK

**Keywords:** Conservation biology, Biodiversity, Biogeography, Conservation biology

## Abstract

Species conservation assessments using the criteria outlined by the *International Union for the Conservation of Nature Red List* can be compromised by limited data availability. Species-rich tropical plant taxa with numerous microendemics are particularly problematic. This study focusses on the *Begonia* flora of the Indonesian island of Sulawesi, comprised of 65 herbaceous species mainly found in rainforest habitats. Sixty-two species are Sulawesi endemics, including 20 species restricted to limestone karst landscapes. Forty-eight species are represented by fewer than 10 herbarium collections. Here, we outline and discuss an approach that, despite these data limitations, allows meaningful conservation assessments by integrating analyses of occurrences, data primarily based on remote sensing approaches, including forest landscape integrity, forest cover loss, and land cover, and extent of suitable habitat estimation. The results indicate that most Sulawesi *Begonia* species are narrow endemics whose rainforest habitats have substantially deteriorated in the last two decades: 27 species are assessed as Critically Endangered, 24 as Endangered, six as Vulnerable, five as Least Concern, and three species are Data Deficient. Conservation action, including extension of the protected area network in Sulawesi with emphasis on areas of old-growth forest and limestone karst landscapes, and strengthening of *ex-situ* living collections, is recommended.

## Introduction

The criteria and categories defined by the *International* *Union for Conservation of Nature (IUCN) Red List* provide a widely recognized framework for evaluating the extinction risk and threat status of species, which has guided conservation efforts for over five decades^1,2^. The primary goal of the *IUCN Red List* is to provide information and analyses on the status, trends, and threats to species to inform and catalyse action for biodiversity conservation^2^. The placement of species in one of the five extinction risk categories—Least Concern (LC), Near Threatened (NT), Vulnerable (VU), Endangered (EN) or Critically Endangered (CR)—is informed by data on population sizes and trends, current species geographic ranges, and observed, inferred, or predicted range or habitat decline^2,3^. In addition to the assessment, the list provides various information on associated aspects such as species habitat and ecology, use and trade, threats, and conservation measures in place. By providing a scientifically rigorous methodology for assessments and a respected online source of extinction risk, the *IUCN Red List* has the potential to influence crucial conservation aspects such as (i) scientific research, (ii) policy and conventions, (iii) planning, (iv) resource allocation, (v) decision-making on the implementation of proposed projects, and (vi) education and raising awareness^1,2^.

While providing a scientifically robust methodology for assessments, the criteria employed by the IUCN can be problematic to apply for taxa that lack robust occurrence data, such as specimens in natural history collections or well-documented field observations. Less than 20 percent of vascular plant species have been assessed at global level and added to the *IUCN Red List* to date (66,113 assessments in Jan 2024^2^), and assessments are taxonomically and regionally biased. Tropical areas remain understudied and underrepresented in biodiversity data and conservation assessments^4,5^. Species-rich tropical taxa are often particularly problematic for extinction risk assessments^6–8^. They represent diverse and characteristic elements of tropical biodiversity and include many rare and potentially threatened species, but effective threat status assessment is frequently hampered by (i) the lack of a stable taxonomic framework and limited knowledge of species circumscriptions; (ii) a preponderance of narrow endemics, many of which occur in remote areas that are difficult to access; and (iii) associated poor occurrence data availability and poor knowledge of species geographic ranges. The dearth of underlying data can preclude the application of assessment criteria related to population sizes and trends, and limits the meaningful assessment of conditions related to geographic ranges, such as area of occupancy estimates (AOO, the area within the geographic range of a species that is actually occupied by the species^3,9^) as well as the use of species-distribution modelling (SDM) approaches to infer potential geographic ranges^8^.

**Figure 1:**
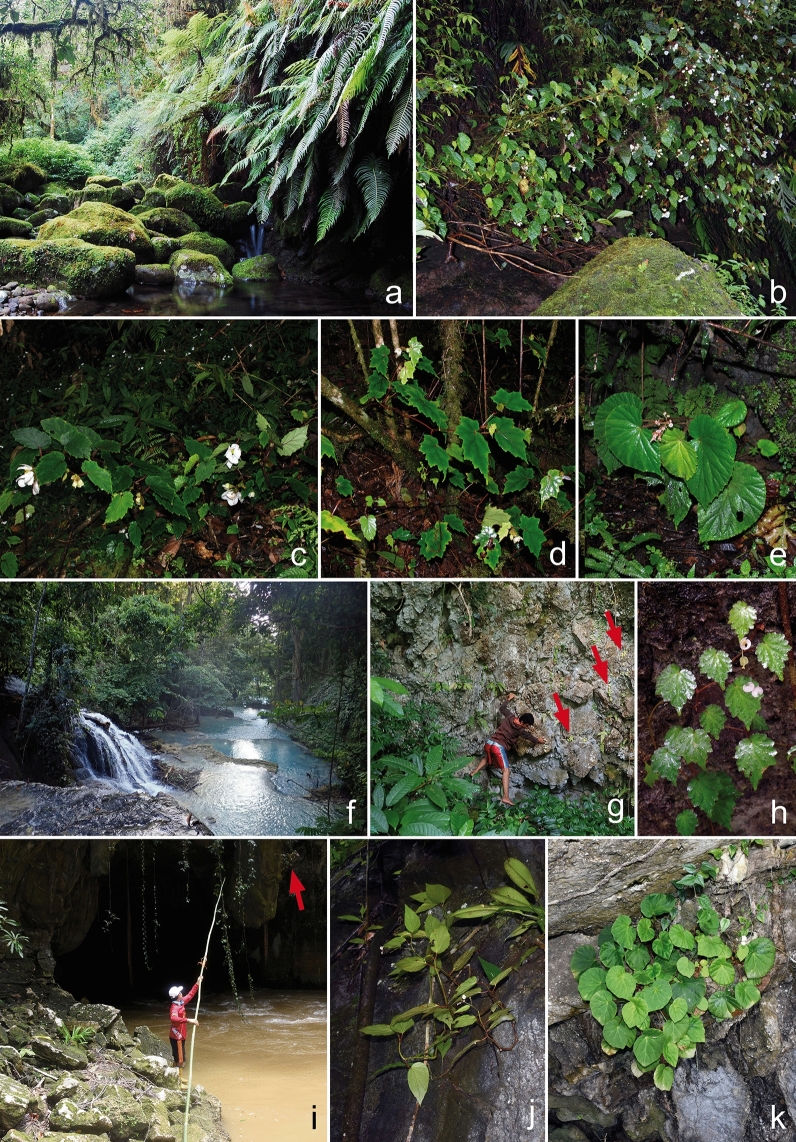
Examples of typical habitats and growth habits of Sulawesi *Begonia* species. (**a**) Stream in primary montane forest on Gunung Bawakaraeng, SW Sulawesi. (**b**) *Begonia bonthainensis* growing terrestrially on the stream bank (wider habitat shown in **a**). (**c**) *Begonia sanguineopilosa* growing terrestrially on the forest floor. (**d**) *Begonia rantemarioensis* growing terrestrially on the forest floor, on a steep slope. (**e**) *Begonia ozotothrix* growing terrestrially on the forest floor, at the base of a limestone boulder. (**f**) Stream on limestone bedrock in lowland forest close to Luwuk, eastern Central Sulawesi. (**g**) Vertical limestone wall at side of stream (wider habitat shown in **f**), red arrows indicate *Begonia willemii* individuals. (**h**) *Begonia willemii* growing lithophytically on a limestone wall (wider habitat shown in **g**). **(i)** Limestone karst landscape with river and cave in Matarombeo, SE Sulawesi; the red arrow indicates *Begonia watuwilensis* growing lithophytically on a stalactite. (**j**) *Begonia watuwilensis* growing lithophytically on limestone. (**k**) *Begonia matarombeoensis* growing lithophytically on a limestone cliff. Photo credit: a, b, f–k, Wisnu H. Ardi; c–e, Daniel C. Thomas.

**Figure 2:**
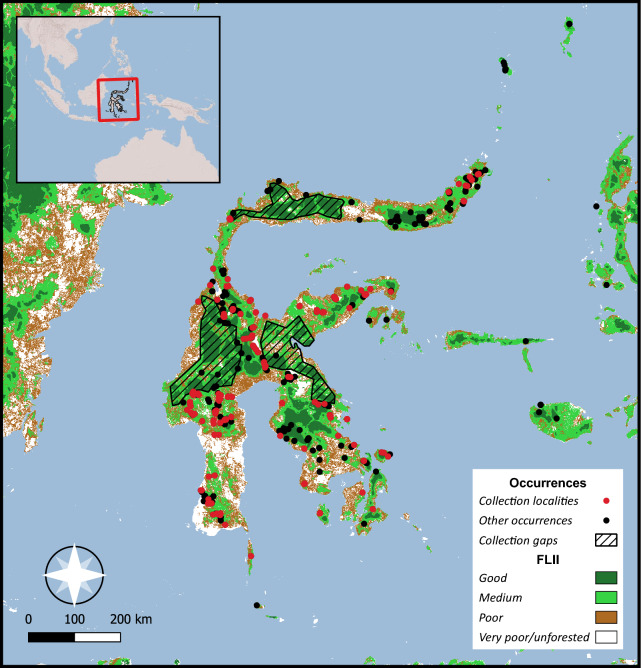
Occurrences of Sulawesi *Begonia* species. Collection localities visited by the authors (W.H. Ardi or D.C. Thomas) are indicated by red dots, all other occurrences from herbarium specimens and observations by black dots. The base map shows categorized forest landscape integrity index (FLII) data^20^.

A prime example of such a problematic species-rich tropical taxon is *Begonia* (Begoniaceae), a mega-diverse (2149 currently accepted species^10^), pantropical genus of herbs and soft-wooded shrubs, which are characteristic elements of the herb layer in tropical rainforest worldwide^11^. The genus shows a preponderance of narrowly distributed species^12^ including many calciphile species that are known from only a single or few limestone outcrops^13–15^. Here, we focus on the *Begonia* flora of the Indonesian island of Sulawesi to better understand the assessment challenges outlined above. Sulawesi is the 11^th^ largest island in the world (ca. 186,404 km^2^) and remains one of the most poorly botanically explored regions in tropical Southeast Asia^16,17^. It forms a major part of the Wallacea biodiversity hotspot and has been frequently highlighted as priority area for global conservation^18,19^. The extend of old-growth forest and intact forest landscapes has changed dramatically in Sulawesi in the last 50 years, and lowland forests have been largely converted or are in poor condition. This is primarily due to commercial logging operations, clearing of forest for agriculture including, since the early 1990s, the establishment of extensive oil palm plantations, development of settlements and other human infrastructure, anthropogenic fires, and mining of nickel and gold^16,20–23^. This trend is continuing and substantial additional forest cover loss in Sulawesi has been predicted for the next 30 years with rates of loss between 2000 and 2053 predicted at 32% in Gorontalo and North Sulawesi, 33% in Southeast Sulawesi, 34% in West and South Sulawesi and 53% in Central Sulawesi^23^. Sulawesi begonias are a diverse and easily recognisable element of primary rainforest and pristine limestone karst landscape habitats in Sulawesi (Fig. 1), and many species are attractive and of considerable horticultural potential. As such, *Begonia* species have all the hallmarks of flagship species for conservation^15^ and could serve as prime examples for conservation education and raising awareness. There is no current revision of the *Begonia* flora of Sulawesi, but extensive field work focussing on *Begonia* collections (Fig. 2) and taxonomic work using material from 23 herbarium collections have put the diversity of Sulawesi *Begonia* into focus: Sixty-five currently accepted *Begonia* species have been reported from the island, 62 of which are endemic to Sulawesi, and 20 of which are restricted to habitats in limestone karst landscapes^24^. Much of this knowledge is from relatively recent studies; forty-seven Sulawesi *Begonia* species were described, and the circumscription and distribution of numerous species were clarified since the year 2000^13,24–46^.

Despite this considerable body of recent taxonomic studies focussing on Sulawesi *Begonia*, some collection gaps remain (Fig. 2) and many species remain rather poorly collected: 12 Sulawesi *Begonia* species are only known from the type collection, 30 species from less than five collections and observations, and 48 species from less than 10 collections and observations (Supplementary Information Table 1). This kind of data scarcity poses major challenges for threat and extinction risk status assessments, and, consequently, taxa are often considered Data Deficient (DD), i.e. the available data is interpreted as to provide “inadequate information to make a direct, or indirect, assessment of its risk of extinction based on its distribution and/or population status”^3^. For example, the preliminary assessments presented in *An Annotated Checklist of Southeast Asian Begonia* regarded ca. 51% of species (266 species) as DD^6^. A study on conservation assessments in the species-rich Neotropical tree genus *Guatteria* (184 species, Annonaceae), which used a species-distribution modelling approach, considered species as Data Deficient (DD) when less than five occurrences were available (61 species or ca. 34% of their assessed species)^8^.

Preliminary conservation assessments of most Sulawesi *Begonia* species were provided in previous publications^6,24^, but 15 species were considered DD and two species have not been previously assessed. Additional herbarium material of many of these species has been collected and some well-documented observational data^47^ have been made available since these preliminary assessments were published, necessitating reassessments of their conservation status. Moreover, most of these preliminary assessments relied on field observations of threats and habitat disturbance from collection trips, and did not consider available data on land cover, forest landscape integrity, forest cover loss, and karst occurrence, i.e. data on crucial parameters that are indicative of the presence of threats (e.g. forest conversion for crop land and human habitation, fire occurrences, presence of managed forest concessions and oil palm plantation concessions) and habitat availability. Disturbance tolerance varies considerably in Sulawesi begonias, but most species are adapted to deep or partial shade and mostly humid conditions in the rainforest herb layer (see habitat descriptions in Supplementary Information Tables 2 and 3, and Fig. 1) and will show sun damage and eventually die when they are permanently and fully sun-exposed. Limestone presence is an obvious prerequisite of habitat availability for the limestone endemic species. Moderate to good forest landscape integrity, i.e. areas that have primary or secondary forest and are not converted or heavily modified for agriculture or human infrastructure, and karst landscape presence can be seen as reasonable proxies for potential habitat availability within the known ranges of Sulawesi *Begonia* species, and understanding the extent of potentially suitable habitats within species ranges can inform conservation status assessments^48^.

Our aim is to understand the extent to which robust conservation assessments are possible in a species-rich tropical group with a preponderance of microendemics. Our objectives are to (i) compile available occurrence data from multiple sources and provide distribution maps of Sulawesi *Begonia* species; (ii) integrate the occurrence data with a suite of environmental data layers (e.g., forest landscape integrity, forest cover loss, land cover, karst occurrence) to maximise insights from the limited data available; (iii) provide new conservation assessments for the endemic *Begonia* species in Sulawesi based on this data; and (iv) build on this experience to outline an approach that can be used on other groups presenting similar challenges.

## Results

### Species geographic ranges

Distribution maps are presented in Supplementary Information Figs. 1–13. Extent of occurrence (EOO), area of occupancy (AOO) and number of location estimates of the 62 assessed species are presented in the Supplementary Information Table 1.

EOOs, AOOs and the number of locations of three species (*Begonia humilicaulis*, *B. ignita* and *B. imperfecta*) could not be estimated as no occurrence data was available, and EOOs of an additional 30 species could either not be estimated as less than three occurrence points were recorded or EOO values were smaller than the AOO of the respective species. Following in the IUCN guidelines, the EOOs of these species were set to the respective AOO values^3^.

Of the 62 assessed Sulawesi *Begonia* species, 40 have EOOs smaller than 100 km^2^ (benchmark for CR status), 11 species have EOOs between 100 km^2^ and 5000 km^2^ (benchmark for EN status), three species have EOOs between 5000 km^2^ and 20,000 km^2^ (benchmark for VU status), and eight species have EOOs larger than 20,000 km^2^.

AOOs of 22 species are below 10 km^2^ (the benchmark of CR status), and AOOs of 40 species are between 10 km^2^ and 500 km^2^ (the benchmark for EN status).

Twenty-nine species were assessed as to occur in only a single location (condition under Criterion B supporting CR status), 25 species were assessed to occur in two to five locations (supporting EN status), five species to occur in six to 10 locations (supporting VU status), and three species to occur in more than 10 locations.

### Area of habitat estimations

Species habitat descriptions and elevational ranges are summarized in Supplementary Information Tables 2 and 3.

Area of habitat (AOH) estimations are summarized in Supplementary Information Table 1. Estimates based on medium to good forest landscape integrity of 30 species are smaller than 10 km^2^ (AOO benchmark for CR status), 15 species have AOHs between 10 and 500 km^2^ (AOO benchmark for EN status), four species have AOHs between 500 and 2000 km^2^ (AOO benchmark for VU status), and 13 species have AOHs larger than 2000 km^2^. AOH estimates based on limestone karst landscape presence within the species ranges of the 20 limestone-endemic species included 10 limestone-endemic species that have AOHs smaller than 10 km^2^, seven species that have estimates between 10 and 500 km^2^, and three that have estimates between 500 and 2000 km^2^ (*Begonia flacca*, *B. ranoposoensis*, and *B. watuwilensis*).

### Forest cover loss

Estimated forest cover loss between 2001 and 2022 within species ranges is summarized in Supplementary Information Table 2. Estimates ranged from 0.4 to 48% (average 10%) forest cover loss within the timeframe.

### Presence in protected areas

Twenty-four of the 62 assessed species were present in at least one protected area. Seven species were present in at least two protected areas. The most species were reported from Bogani Nani Wartabone National Park (IUCN category II, 6 species), Lore Lindu National Park (IUCN category II, 5 species), Gunung Sojol Nature Reserve (IUCN category Ia, 4 species) and the Nantu Wildlife Reserve (IUCN category III, 4 species) (Supplementary Information Table 4).

### Conservation category assessments

**Figure 3:**
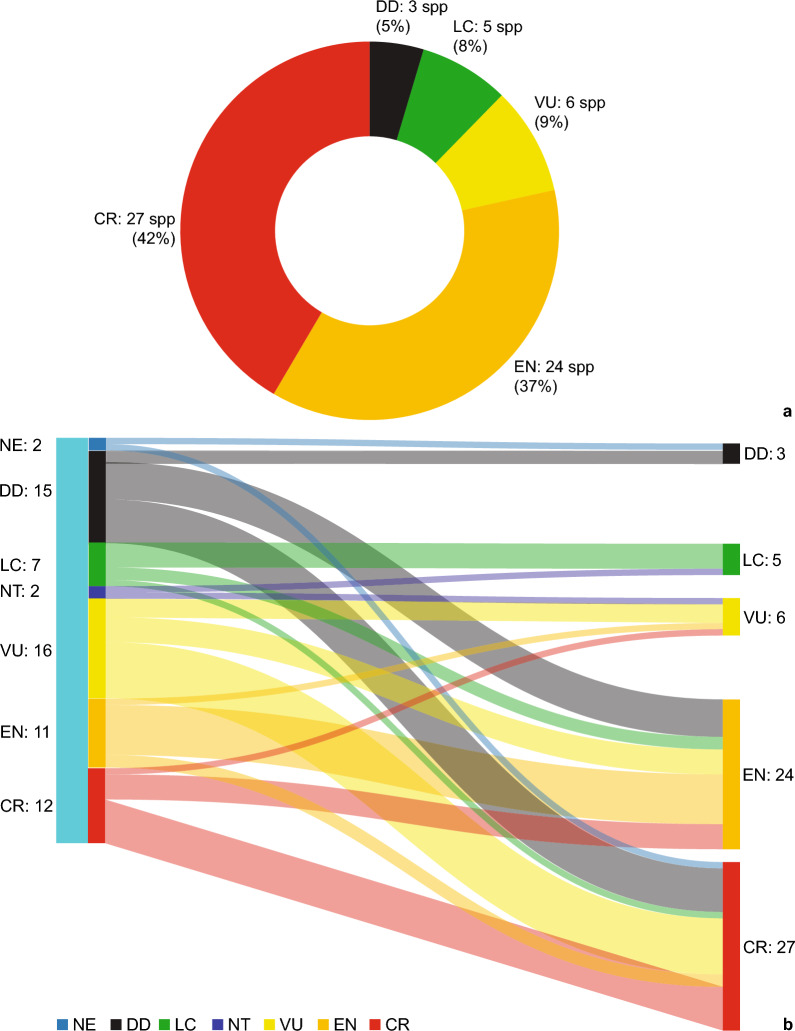
Summary of conservation status assessments and assessment changes for 65 Sulawesi *Begonia* species. (**a**) Doughnut chart summarizing proposed IUCN assessments. (**b**) Sankey diagram indicating assessment changes (previously published preliminary assessments on the left and current proposed assessments on the right). *LC* least concern, *DD* data deficient, *EN* endangered, *NE* not evaluated, *NT* near threatened, *VU* vulnerable.

Conservation category assessments are presented in Supplementary Information Table 1, and current assessments and changes in comparison to previously published preliminary assessments are summarized in Fig. 3. Using our approach and currently available data, the number of species considered DD declined from 15 to three. There were marked increases in level of extinction threat for 19 species, with the number of species assessed as EN rising from 11 to 24, and the number of CR species rising from 12 to 27.

Three species whose ranges are unknown and for which no occurrence data was available, were considered Data Deficient (DD): *Begonia humilicaulis*, *B. ignita*, *B. imperfecta*.

Five species were assessed as Least Concern (LC): *Begonia aptera*, *B. mendumiae, B. ozotothrix, B. rieckei, B. robusta*. These include the only three species in the dataset whose ranges extend beyond Sulawesi, and two species (*Begonia mendumiae* and *B. ozotothrix*) that have wide ranges on Sulawesi and relatively wide habitat availability within their ranges. Both these species also have relatively wide tolerances to habitat disturbance (see habitat descriptions in the Supplementary Information).

Six species, including four limestone-endemic species, were assessed as Vulnerable (VU): *Begonia flacca, B. hooveriana, B. labenkiensis, B. mekonggensis, B. sidolensis, B. willemii*. Two of these species (*B. labenkiensis, B. sidolensis*) show very restricted ranges and single locations, but are from areas that show no signs of anthropogenic disturbance and only minor forest cover loss since 2001. The other four species are more widespread but have EOOs smaller than 20,000 km^2^ and less than 10 locations, and observation of threats and inferred continuous habitat decline within their range resulted in a Vulnerable assessment.

Twenty-four species were assessed as Endangered (EN), including nine of the limestone-endemic species. These species are known from only two to four locations and EOOs of 22 of these species are smaller than 2000 km^2^ (benchmark for EN under Criterion B is > 5000 km^2^). Exceptions are *Begonia comestibilis* and *B. cuneatifolia*, which have relatively large EOOs (> 20,000 km^2^), but are only known from four and three locations, respectively. Most of the species in this category show at least some collections from contiguous areas of medium or good forest landscape integrity.

Twenty-seven species, including seven limestone-endemic species, were assessed as Critically Endangered (CR). All are only known from single locations and have EOOs smaller than 100 km^2^. Collection localities of most species in this category are either in areas that show poor forest landscape integrity or from forest margins or small forest fragments of moderate forest landscape integrity near areas cleared for agriculture or human settlements.

## Discussion

Our integrative approach allowed complete assessments of 62 of the 65 Sulawesi *Begonia* species, decreasing the number of species that were not assessed or previously considered Data Deficient from 17 to three. The results indicate marked increases in the level of extinction threat for 19 species, and a large percentage of species were assessed in the threatened categories VU (6 species), EN (24 species) and CR (27 species). Most Sulawesi *Begonia* species are found in primary and secondary forest habitats, and our findings are in line with the dramatic forest cover loss associated with changes in land use in Sulawesi over the last 50 years^16,20–23^. Only a minority of species has been reported from any protected area. Clearly, additional conservation action is required to preserve these species including extension of the protected area network in Sulawesi with emphasis on areas of old-growth forest and limestone karst landscapes^16,49^, and funding of *ex-situ* conservation of rare *Begonia* species such as in the living collections at Bali and Bogor Botanic Gardens^50^.

The *IUCN Red List* is assumed to influence crucial conservation aspects^1^, but for many tropical regions, assessments of only a small percentage of biota have been published, e.g. *Plants of the World Online* (https://powo.science.kew.org, accessed January 2024) lists 4,864 vascular plant species from Sulawesi, but the *IUCN Red List* includes only 678 assessments (ca. 14% of Sulawesi’s vascular plant species^2^). Considerable additional efforts are needed to fill this void to create an effective basis to inform policy and decision-making, which remains crucial given ongoing and predicted deforestation on the island^23^. Tropical areas remain understudied and underrepresented in biodiversity data and conservation assessments^4,5^, and prerequisites for conservation assessments such as (i) a sound taxonomic basis including baseline work clarifying species circumscriptions and distributions, and (ii) extensive fieldwork and collections from relevant habitats, are often not fulfilled. For Sulawesi *Begonia* there is a relatively sound basis, but some conspicuous collection gaps remain, including some extensive areas of suitable forest habitats in western central Sulawesi and along the northern arm of the island, as well as areas with extensive limestone karst landscapes in eastern central Sulawesi. However, the localities visited by the authors (W.H. Ardi and D.C. Thomas) span suitable habitats across most of the island and elevational gradients (Fig. 2). Collections from this field work have clarified distributions of numerous species that were originally described from very limited material, e.g. *Begonia ozotothrix* was described from a few collections from limestone forest on the eastern arm of Central Sulawesi^31^, but is now known to be widespread on the island (EOO > 84,000 km^2^) and to also occur on granite and alluvial substrates (Supplementary Information Table 3); *B. flacca* was only known from a few historical collections^35^, but is now known to be widespread in Southeast Sulawesi (EOO > 16,000 km^2^); *B. hooveriana* was described from a single, strongly disturbed urban locality^33^, but is now known to have an EOO of more than 10,000 km^2^. This indicates that the current collection density allows to effectively identify taxa that are more widespread on the island. Additional collections efforts will undoubtedly identify numerous new species, further clarify species distributions, and show that geographic ranges of some species are currently underestimated, but it seems unlikely that many putatively microendemic *Begonia* species will turn out to have wide distributions on Sulawesi. Nevertheless, the limitation of a sparse data basis and the caveat that additional exploration may reveal wider geographic ranges or additional locations, should be and was explicitly indicated in the proposed IUCN assessments.

In addition to dedicated taxonomic research and field work, an important role in adding data and clarifying species distributions also comes from observations by citizen scientists submitted to publicly available data portals such as *iNaturalist*^47^. Well-documented, verifiable Sulawesi *Begonia* observations are still sparse but include crucial observations of narrowly distributed species such as *B. insularum*, *B. hispidissima* and *B. matarombeoensis* that were previously only known from a few herbarium collections.

The compilation of and comparison with previously published preliminary assessments^6,13,27,28,30–32,34–36,38–46^ indicate numerous changes of assigned threat categories in the current assessments (Fig. 3). Direct comparison is not always possible, as the data basis for many assessments has changed over time, but multiple reasons can be identified: (i) additional occurrence data availability; (ii) different criteria used to estimate the number of locations or different interpretations of severe range fragmentation; (iii) range and habitat decline assessments not utilising forest cover loss, forest landscape integrity and land cover data; (iv) assessments not considering habitat availability within the known range; and (v) different interpretation or erroneous application of the *IUCN Red List* criteria and conditions.

Previous assessments did not use a consistent method to estimate the number of locations or assess severe range fragmentation—a crucial element when applying criterion B (geographic range) to evaluate whether a taxon belongs in a threatened category. The rationale underlying location number estimation, clarifying what plausible threats were considered and what data is available to determine the spatial and temporal scales of the impacts of the respective threats, was not given. This can have a major effect on the outcome of an assessment and is also required to guarantee reproducibility of the results. Similarly, when using the ‘severely fragmented’ range condition, data supporting this assessment, e.g. from population genetic studies, should be indicated.

The integration of forest cover loss and land cover data in the assessments allowed a better evaluation of threats and the likelihood of continuing range and habitat decline. Direct observations of threats and habitat loss in the field are valuable but often limited by the frequency of field work and the size of the areas surveyed. When working with taxa that are largely restricted to tropical forest habitats, additional sources that can add to these assessments can come from (i) landcover data^51^ and forest landscape integrity index data^20^ indicating whether collection localities are in intact forest landscapes or encroached by land cleared for agriculture and human settlements; (ii) forest cover loss data^21^ that can indicate how forest habitats have been faring in the twenty-first century and give insights into the role of specific threats such as forest fires^52^; and (iii) available data on land use involving managed forest, oil palm and mining concessions^53,54^ can give additional insights into specific anthropogenic threats and the likelihood of habitat loss in the future. Curated data sets on forest cover loss and land use as well as some simple analysis tools made available by *Global Forest Watch* (www.globalforestwatch.org) can be easily employed to improve threat and habitat loss assessments in the IUCN framework. Remote-sensing data can also corroborate field observations indicating the absence of anthropogenic threats. While at least some collection localities of most species were in areas where forest has been converted for agriculture or human infrastructure, in small forest fragments surrounded by or in forest margins near such areas, some exceptions could also be identified. *Begonia labengkiensis*, for example, is from a small island off Southeast Sulawesi, where no signs of anthropogenic disturbance were seen during field work^46^ and only minimal forest cover loss is indicated by remote sensing^21,52^ and landcover data^51^. This species, adopting a cautionary attitude because of the very restricted range, a single location, and the limited number of observed mature individuals, was assessed as Vulnerable (VU D2) as it may be prone to the effects of human activities (e.g. anthropogenic forest fires for forest clearing for agriculture or tourist infrastructure) or stochastic events within a very short time period in an uncertain future^3^.

The limited available occurrence data for many Sulawesi begonias can limit a meaningful application of some aspects related to geographic ranges such as area of occupancy (AOO) estimation. AOO is usually calculated using presence data from herbarium specimens by overlaying the occurrence points with a 2 × 2 km grid and calculating the total area of occupied grid cells. This implies that at least 500 collections from unique grid cells are needed to surpass the benchmark for VU status (AOO < 2000 km^2^), which is not given for any of the 65 Sulawesi *Begonia* species. AOO values of true microendemics are still valuable for assessments, but for more widely distributed but relatively poorly collected species, AOO values will be underestimates of the occupied area and indicate an overestimated extinction risk. For the latter category of species, estimates of the area of available habitat (AOH) within a species range may give some additional insights, as AOH can be interpreted as an upper bound of the potential AOO of a species^48^. This can inform conservation assessments, e.g. the limestone-endemic species *Begonia willemii* shows a relatively wide EOO in Sulawesi (> 23,000 km^2^), but limited presence data from 12 herbarium specimens indicates a certainly underestimated AOO of only 32 km^2^. An estimated AOH of 428 km^2^ based on the presence of strongly fragmented patches of lowland to hill forest in limestone karst landscapes at the suitable elevational range within the EOO, in combination with only nine known localities and evidence of considerable recent and likely continuing forest cover loss in the EOO (12% between 2001 and 2022), indicate that this taxon should be considered Vulnerable. In contrast to this, *Begonia mendumiae* has both a relatively wide EOO (66,842 km^2^) and AOH (9398 km^2^) based on forested areas that have medium to good forest landscape integrity in the EOO, indicating that likely both the AOO of 52 km^2^ and only 10 known locations are underestimates and that further botanical exploration will likely result in the discovery of multiple additional localities. Accordingly, this species was assessed as Least Concern.

In addition or as alternative to AOH estimation, other studies have employed species-distribution modelling (SDM) approaches to estimate species geographic in the face of limited data availability (e.g.^8,55^) and to overcome some limitations of presence-only data from herbarium specimens such as sample bias towards more accessible areas^56^, more easily accessible taxa, or more attractive ‘biodiversity hotspot’ areas^16^ (see discussion in Verspagen & Erkens^8^). An SDM approach may be also provide valuable insights on Sulawesi begonias, but the occurrence data basis is still too sparse for many species (30 species are known from less than five occurrences). Moreover, the data usually underlying SDM, such as extrapolated climatic data sets and digital elevational models, may be insufficient to capture some crucial factors. Begonias often occupy very specific microhabitats including lithophytic species growing directly on rock or in rock crevices, and others are primarily found in shady, humid habitats or along stream and river banks or at waterfalls (Fig. 1; see habitat descriptions in Supplementary Information Tables 2 and 3). Moreover, data on biological factors such as dispersal capabilities are rarely integrated in SDM approaches, which can lead to overestimates of geographic ranges^57^. Consequently, extensive subsequent ground truthing would still be required to confirm the results of SDM approaches for Sulawesi begonias.

The *Guidelines for Using the IUCN Red List Categories and Criteria* state that the “absence of high-quality data should not deter attempts at applying the criteria”^3^. This is relevant for *Begonia*, one of the fastest growing genera of flowering plants^11,58^. Numerous new *Begonia* species descriptions are published every year—39 species in 2023 alone (*International Plant Index* data: https://ipni.org/)—frequently based on very limited material. From our experience with Sulawesi *Begonia* assessments, we can derive several recommendations for effective conservation status assessment of begonias and similar species-rich tropical groups with a preponderance of microendemics: (i) Occurrence data from not only specimens in natural history collections but also well-documented observations should be considered for range estimation. (ii) When using criterion B (geographic range) and location number estimation, the underlying rationale and considered plausible threats should be explicitly stated. (iii) In addition to field observations, integration of other data layers such as land cover, forest cover loss and forest landscape integrity, can provide insights into threats and the likelihood of continuing range and habitat decline. (iv) If proxies for suitable habitats can be determined, e.g. by considering edaphic conditions such as the presence of limestone karst landscapes or forest quality indicated by the forest landscape integrity index^20^, then AOH estimation should be employed. This provides an upper boundary for the extend of the AOO in the known range, potentially providing valuable new insight to inform conservation status assessments.

The integrative approach outlined here allows assessments of most species of the diverse Sulawesi *Begonia* flora in the face of data availability limitations. However, this approach is only feasible when some prerequisites regarding taxonomic baseline work are met, emphasizing the importance of funding of taxonomic revisionary work, field work focussing on specimen collecting and surveying populations, mobilising data on tropical taxa, e.g. checklist preparation and digitizing both larger and smaller regional herbarium collections, and supporting citizen science initiatives including observation databases such as *iNaturalist*. The identified collections gaps, including extensive areas of good forest landscape integrity and limestone karst areas, should be prioritised for expeditions to gain further insights into the diversity, distributions and conservation status of the *Begonia* flora of Sulawesi.

## Methods

### Data selection

The *Begonia* flora of Sulawesi includes 65 currently accepted species, 95% of which (62 species) are endemic to the island^24^. Occurrence data was compiled from (i) the study of herbarium collections (A, B, BAS, BM, BO, C, CEB, E, FI, FIPIA, HAST, K, KRB, L, MICH, NY, P, PNH, S, SING, U, WAG, WAN; herbarium codes following Thiers^59^) including georeferenced herbarium specimen data and images made available in the *Begonia Resource Center*^10^; and (ii) observations with associated GPS data and photos from *iNaturalist*^47^, our own observations and observations provided by P. Blanc, A. Bour, and R.P.P. Ahmand. All specimen and observation identifications were made or confirmed by D.C. Thomas and W.H. Ardi, and data of 1498 herbarium specimens from 915 unique collections, and 43 observations, including 694 occurrences, were compiled (see Supplementary Information Table 1).

The occurrence point localities were plotted in *QGIS 3.28.3 Firenze*^60^ together with administrative boundaries (Indonesia, Sulawesi, Sulawesi provinces using GADM data^61^). Other data employed in the framework of the conservation assessments included SRTM 1 arc-second digital elevation model data^62^, limestone karst landscape distribution data^63^, protected areas^64^, forest landscape integrity index data^20^, forest cover loss data^21,52^ and Indonesian landcover data^51^.

Species habitat data and elevational ranges were compiled from field observations, herbarium label data, locality elevations extracted from the SRTM 1 arc-second digital elevation model data^62^ and the literature^13,24–46^.

Previously published, preliminary conservation assessments were compiled from the literature^6,13,27,28,30–32,34–36,38–46^.

### Red list assessments

**Figure 4:**
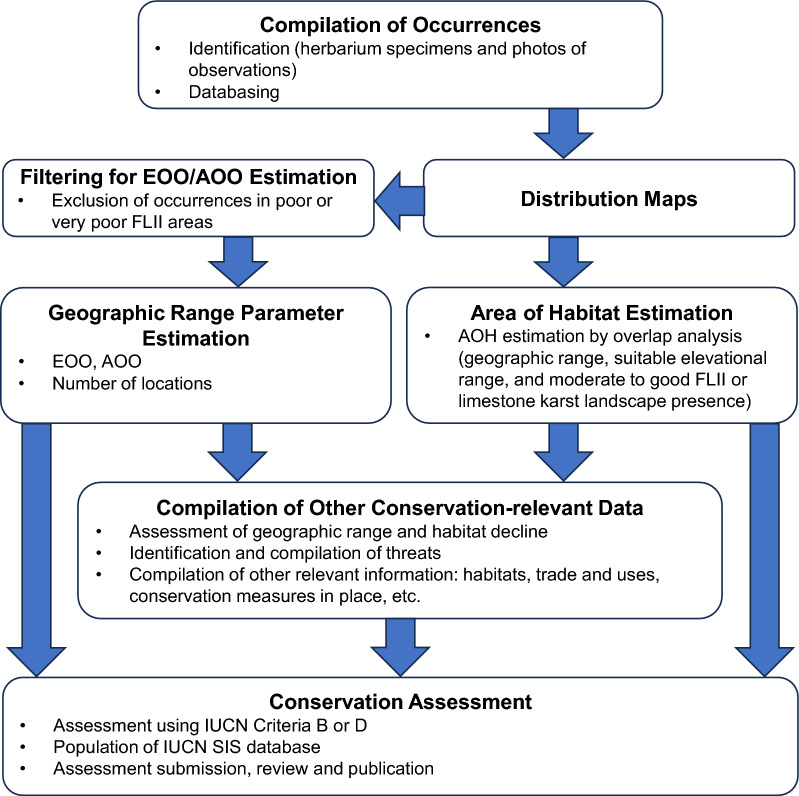
Workflow overview. *AOH* area of habitat, *AOO* area of occupancy; *EOO* extent of occurrence, *FLII* Forest Landscape Integrity Index.

In the absence of detailed population size and trend data, our Red List assessments focussed primarily on Criterion B (geographic range in the form of extent of occurrence [EOO] and/or area of occupancy [AOO]). Criterion D (very small or restricted populations) was used in two cases. An overview of the workflow is presented in Fig. 4.

The occurrence data was filtered for both Extent of Occurrence (EOO) and Area of Occupancy (AOO) estimations. Both of these geographic range parameters focus on areas presently occupied by a taxon: EOO is the area contained within the shortest imaginary boundary (minimum convex polygon) which can be drawn to encompass all known, inferred or projected sites of present occurrence of a taxon, while AOO is the area within the EOO that is actually occupied by the taxon (usually measured by overlaying a 2 × 2 km grid and adding the total area of occupied cells)^9^. Occurrence data from recent collections or observations (collected since 1993) and historical collections (before 1993) whose AOO grid cells show at least some areas of medium to good forest landscape integrity^20^ were used for EOO and AOO calculations. This effectively filters out occurrences from historical collections from areas that were cleared or heavily modified for agriculture or human infrastructure as indicated by AOO grid cells that are completely covered by very poor or poor forest landscape integrity areas. Forest landscape integrity was used as a proxy indicating whether there is a high likelihood that the species still occurs in the area, as most Sulawesi begonias occur in primary and secondary forest habitats (see Introduction and Supplementary Information Tables 2 and 3). EOO and AOO estimations were performed using *GeoCAT*^65^.

To estimate the Area of Habitat (AOH), i.e. the extent of suitable habitat available to a species within its range^48^, species distributional and elevational ranges (presence in elevational bands based on Sulawesi forest elevational categories used by Cannon et al.^16^: lowland forest, 0–400 m; hill forest, 400–850 m; upland forest, 850–1500 m; montane forest, 1500–2500 m; tropalpine, 2500 m upwards) were determined from the compiled occurrences and herbarium label data. We overlayed the geographic range, SRTM 1 arc-second digital elevation model data^62^, and categorized forest landscape integrity data^20^ (forest landscape integrity index data categorized in very poor/unforested, poor, medium and good integrity, see Fig. 2). The geographic range was either the known species distribution, or, if less than three occurrences were known or the distribution area was smaller than the AOO, the AOO of the species. Subsequently, an overlap analysis was performed, estimating the area in the geographic range that shows a suitable elevational range and medium to good forest landscape integrity^20^. For 20 limestone-endemic species (see Supplementary Information Tables 2 and 3), presence of limestone karst landscape^63^ was used as a proxy for potential habitat availability instead of forest landscape integrity data to determine the AOH.

The number of locations—in the *IUCN Red List* framework defined as geographically or ecologically distinct area in which a single threatening event can rapidly affect all individuals of the taxon^9^—was determined by considering the impact of plausible threats with large-scale effects such as forest fires or commodity-driven deforestation (e.g. oil palm plantation concessions or timber harvesting in managed forest concessions). Based on data showing the extend of forest loss due to fire^52^ and the size of managed forest concessions and oil palm plantation concessions on Sulawesi^53,54^, only localities that were separated by at least 20 km were considered separate locations, i.e. collections present in a 20 × 20 km area were counted as being present in a single location.

To determine threats and the likelihood for continuing range and habitat loss, we compiled notes of direct observations of habitat disturbances from extensive field work in Sulawesi (Fig. 2), and estimated the amount of forest cover loss between 2001 and 2022 within the EOO of each species using total forest cover loss^21^ and forest cover loss from fire data^52^. We also overlayed Indonesian landcover data^51^ and forest landscape integrity index data^20^, and if AOOs were overlapping with or in close proximity (< 2 km buffer) to areas that show poor forest landscape integrity, where forest has been converted for agriculture or human infrastructure or were fires occurred, these aspects (agriculture, human habitation, fires) were noted as potentially ongoing threats. If considerable tree cover loss (> 1% of forested area) and threats that are likely ongoing were identified, this was interpreted as indicative of a high likelihood of continuing habitat and range loss.

Species presence in legally protected areas was determined by mapping protected areas from the *World Database on Protected Areas*^64^.

Compiled data was used to populate the *IUCN Species Information Service* (SIS) database and submitted to IUCN for review.

### Supplementary Information

Supplementary Information.

## Data Availability

*GeoCat* project files and occurrence data are available from the corresponding author on reasonable request and have been added to the Plants_Begonia_Sulawesi_2023 working set in the *IUCN Species Information Service* database (https://www.iucnredlist.org/assessment/sis). The maps in this study, including in the Supplementary Information, were produced with *QGIS 3.28.3 Firenze* (https://qgis.org/en/site/). Sulawesi land cover, forest loss and forest and oil palm plantation concession layers can be found at https://www.globalforestwatch.org/map/. Limestone karst data can be accessed through https://geoportal.esdm.go.id/geologi/. The Forest Landscape Integrity Index data can be found online at https://www.forestlandscapeintegrity.com/. The boundaries of protected areas can be found at https://www.protectedplanet.net/country/IDN. SRTM 1 arc-second digital elevation model data can be found at https://lpdaac.usgs.gov/products/srtmgl1v003/. *iNaturalist* observation data can be accessed through https://www.inaturalist.org.
