# Supplementary material for: Conservation status assessments of species-rich tropical taxa in the face of data availability limitations: insights from Sulawesi Begonia
Source: Sci Rep. 2024 Jun 18;14:14007. doi: 10.1038/s41598-024-64319-7 (PMC11189526; doi:10.1038/s41598-024-64319-7)
Supplement: Supplementary file 1 — Supplementary Information. [file 41598_2024_64319_MOESM1_ESM.pdf]

**Supplementary Information**

# Conservation Status Assessments of Species-rich Tropical Taxa in the Face of Data Availability Limitations: Insights from Sulawesi *Begonia*


Daniel C. Thomas[1,2,*], Wisnu H. Ardi[3,4], Yu Hong Chong[2], Philip Thomas[5] & Mark Hughes[5]

[1]National Parks Board, Singapore Botanic Gardens, Singapore

[2]Nanyang Technological University, School of Biological Sciences, Singapore

[3]Research Center for Biosystematics and Evolution, National Research and Innovation Agency, BRIN, Indonesia

[4]University of Florida, Institute of Food and Agricultural Sciences, USA

[5]Royal Botanic Garden Edinburgh, Scotland, UK

*Corresponding author, email: Daniel_thomas@nparks.gov.sg


**Supplementary Information Table 1.** Occurrence data, geographic ranges (EOO, AOO), area of habitat (AOH) and proposed IUCN assessments.

| | **Herbarium specimens** [#] | **Obser-vations** [#] | **Occur-rences recorded** [#] | **Point lo-calities used in analyses** [#] | **EOO** [km²] | **AOO** [km²] | **Lo-ca-tions** [#] | **AOH1** [km²] | **AOH2** [km²] | **IUCN as-sess-ment** [cate-gory] | **IUCN assessment** [criterion and condi-tions] |
|---|---|---|---|---|---|---|---|---|---|---|---|
| *B. aptera* | 151 | 7 | 121 | 102 | 708948 | 352 | 30-40 | 150231 | -- | LC | |
| *B. balgooyi* | 4 | 0 | 4 | 4 | 2680 | 16 | 3 | 2322 | 197 | EN | B1ab(i,ii,iii)+2ab(i,ii,iii) |
| *B. batusangiensis* | 4 | 0 | 4 | 4 | 8* | 8 | 1 | 0.6 | 0.6 | CR | B1ab(iii)+2ab(iii) |
| *B. bonthainensis* | 12 | 0 | 12 | 10 | 73 | 28 | 1 | 53 | -- | CR | B1ab(i,ii,iii) |
| *B. capituliformis* | 6 | 0 | 6 | 6 | 1354 | 16 | 3 | 700 | -- | EN | B1ab(i,ii,iii)+2ab(i,ii,iii) |
| *B. carnosa* | 4 | 0 | 4 | 2 | 8* | 8 | 2 | 4.5 | -- | EN | B1ab(iii)+2ab(iii) |
| *B. celebica* | 16 | 0 | 16 | 15 | 899 | 24 | 3 | 732 | -- | EN | B1ab(i,ii,iii)+2ab(i,ii,iii) |
| *B. chiasmogyna* | 6 | 0 | 6 | 6 | 1417 | 20 | 3 | 589 | -- | EN | B1ab(i,ii,iii)+2ab(i,ii,iii) |
| *B. comestibilis* | 13 | 0 | 13 | 11 | 23708 | 28 | 4 | 14013 | -- | EN | B2ab(i,ii,iii) |
| *B. cuneatifolia* | 5 | 0 | 4 | 3 | 21720 | 12 | 3 | 6049 | -- | EN | B2ab(i,ii,iii) |

| | | | | | | | | | | | |
|---|---|---|---|---|---|---|---|---|---|---|---|
| *B. didyma* | 3 | 0 | 3 | 3 | 73 | 12 | 1 | 49 | -- | CR | B1ab(i,ii,iii) |
| *B. enoplocampa* | 3 | 0 | 2 | 1 | 4* | 4 | 1 | 3.6 | -- | CR | B1ab(iii)+2ab(iii) |
| *B. flacca* | 20 | 0 | 20 | 17 | 16236 | 32 | 8 | 2699 | 813 | VU | B1ab(i,ii,iii)+2ab(i,ii,iii) |
| *B. gambutensis* | 4 | 0 | 3 | 2 | 8* | 8 | 2 | 4 | -- | EN | B1ab(iii)+2ab(iii) |
| *B. gemella* | 4 | 0 | 2 | 2 | 4* | 4 | 1 | 3 | 2.4 | CR | B1ab(iii)+2ab(iii) |
| *B. guttapila* | 1 | 0 | 1 | 1 | 4* | 4 | 1 | 0.3 | -- | CR | B1ab(iii)+2ab(iii) |
| *B. hekensis* | 5 | 0 | 5 | 5 | 12* | 12 | 1 | 7.7 | -- | CR | B1ab(i,ii,iii) |
| *B. hispidissima* | 5 | 1 | 5 | 5 | 12* | 12 | 1 | 5.5 | -- | CR | B1ab(i,ii,iii) |
| *B. hooveriana* | 19 | 7 | 24 | 24 | 10490 | 80 | 7 | 7814 | 146 | VU | B1ab(i,ii,iii)+2ab(i,ii,iii) |
| *B. humilicaulis* | 1 | 0 | 1 | 0 | -- | -- | -- | -- | -- | DD | |
| *B. ignita* | 1 | 0 | 1 | 0 | -- | -- | -- | -- | -- | DD | |
| *B. imperfecta* | 1 | 0 | 1 | 1 | -- | -- | -- | -- | -- | DD | |
| *B. incudiformicarpa* | 5 | 0 | 3 | 2 | 8* | 8 | 2 | 3.8 | 6.2 | EN | B1ab(iii)+2ab(iii) |
| *B. insueta* | 1 | 0 | 1 | 1 | 4* | 4 | 1 | 0.4 | -- | CR | B1ab(iii)+2ab(iii) |
| *B. insularum* | 2 | 3 | 5 | 5 | 20* | 20 | 1 | 20 | -- | CR | B1ab(i,ii,iii) |
| *B. iskandariana* | 7 | 0 | 6 | 3 | 51 | 12 | 1 | 39 | 15 | CR | B1ab(i,ii,iii) |
| *B. johntanii* | 1 | 0 | 1 | 1 | 4* | 4 | 1 | 0.4 | -- | CR | B1ab(iii)+2ab(iii) |
| *B. kabaenensis* | 1 | 0 | 1 | 1 | 4* | 4 | 1 | 0.6 | 0.6 | CR | B1ab(iii)+2ab(iii) |
| *B. kinhoi* | 4 | 0 | 4 | 4 | 79 | 12 | 2 | 69 | -- | EN | B1ab(i,ii,iii)+2ab(i,ii,iii) |
| *B. labengkiensis* | 3 | 0 | 3 | 3 | 4* | 4 | 1 | 1.8 | 1.9 | VU | D2 |
| *B. lasioura* | 4 | 0 | 4 | 4 | 37 | 12 | 2 | 27 | -- | EN | B1ab(i,ii,iii)+2ab(i,ii,iii) |
| *B. mabberleyana* | 6 | 0 | 5 | 5 | 73 | 12 | 2 | 11 | 28 | EN | B1ab(i,ii,iii)+2ab(i,ii,iii) |
| *B. macintyreana* | 3 | 0 | 2 | 2 | 4* | 4 | 1 | 2.6 | -- | CR | B1ab(iii)+2ab(iii) |
| *B. masarangensis* | 4 | 0 | 4 | 4 | 12* | 12 | 1 | 8.4 | -- | CR | B1ab(i,ii,iii) |
| *B. matarombeoensis* | 4 | 3 | 7 | 7 | 313 | 24 | 2 | 99 | 105 | EN | B1ab(i,ii,iii)+2ab(i,ii,iii) |
| *B. mattampensis* | 3 | 0 | 3 | 2 | 4* | 4 | 1 | 0 | 0 | CR | B1ab(iii)+2ab(iii) |
| *B. medicinalis* | 3 | 0 | 2 | 2 | 8* | 8 | 2 | 5.6 | -- | EN | B1ab(iii)+2ab(iii) |
| *B. mekonggensis* | 16 | 0 | 14 | 14 | 9669 | 48 | 4 | 2319 | -- | VU | B1ab(i,ii,iii)+2ab(i,ii,iii) |
| *B. mendumiae* | 15 | 0 | 14 | 14 | 66842 | 52 | 10 | 9398 | -- | LC | |
| *B. nobmanniae* | 4 | 0 | 4 | 4 | 12* | 12 | 1 | 6.2 | -- | CR | B1ab(i,ii,iii) |
| *B. ozotothrix* | 23 | 3 | 24 | 18 | 84383 | 56 | 8 | 18562 | 7265 | LC | |
| *B. pitopangii* | 1 | 0 | 1 | 1 | 4* | 4 | 1 | 1.3 | -- | CR | B1ab(iii)+2ab(iii) |
| *B. prionota* | 9 | 0 | 9 | 9 | 73 | 20 | 2 | 26 | 5 | EN | B1ab(i,ii,iii)+2ab(i,ii,iii) |
| *B. rachmatii* | 11 | 0 | 10 | 10 | 589 | 24 | 3 | 390 | -- | EN | B1ab(i,ii,iii)+2ab(i,ii,iii) |
| *B. ranoposoensis* | 9 | 0 | 9 | 9 | 1844 | 28 | 2 | 968 | 899 | EN | B1ab(i,ii,iii)+2ab(i,ii,iii) |
| *B. rantemarioensis* | 10 | 1 | 11 | 8 | 35 | 20 | 1 | 30 | -- | CR | B1ab(i,ii,iii) |
| *B. rieckei* | 219 | 4 | 150 | 60 | 5205503 | 220 | 30-40 | 504826 | -- | LC | |
| *B. robusta* | 79 | 0 | 39 | 39 | 550652 | 68 | 11 | 2187 | -- | LC | |

| | | | | | | | | | | | |
|---|---|---|---|---|---|---|---|---|---|---|---|
| *B. rolandfadlii* | 1 | 0 | 1 | 1 | 4* | 4 | 1 | 1.3 | -- | CR | B1ab(iii)+2ab(iii) |
| *B. sanguineopilosa* | 8 | 0 | 8 | 8 | 12* | 12 | 1 | 9.8 | -- | CR | B1ab(i,ii,iii) |
| *B. sarasinorum* | 6 | 0 | 6 | 5 | 8* | 8 | 1 | 1.6 | -- | CR | B1ab(iii)+2ab(iii) |
| *B. siccacaudata* | 20 | 7 | 24 | 22 | 888 | 60 | 4 | 270 | 319 | EN | B1ab(i,ii,iii)+2ab(i,ii,iii) |
| *B. sidolensis* | 1 | 0 | 1 | 1 | 4* | 4 | 1 | 4 | -- | VU | D2 |
| *B. siregarii* | 6 | 0 | 6 | 6 | 14 | 12 | 1 | 0.3 | 6 | CR | B1ab(i,ii,iii) |
| *B. sojolensis* | 3 | 0 | 3 | 3 | 267 | 12 | 2 | 71 | -- | EN | B1ab(i,ii,iii)+2ab(i,ii,iii) |
| *B. sphenocarpa* | 5 | 0 | 4 | 4 | 8* | 8 | 1 | 5.6 | -- | CR | B1ab(iii)+2ab(iii) |
| *B. stevei* | 1 | 0 | 1 | 1 | 4* | 4 | 1 | 2.1 | -- | CR | B1ab(iii)+2ab(iii) |
| *B. strachwitzii* | 6 | 0 | 6 | 6 | 119 | 12 | 2 | 67 | -- | EN | B1ab(i,ii,iii)+2ab(i,ii,iii) |
| *B. tjiasmantoi* | 1 | 0 | 1 | 1 | 4* | 4 | 1 | 2.1 | -- | CR | B1ab(iii)+2ab(iii) |
| *B. torajana* | 8 | 3 | 11 | 11 | 34 | 12 | 2 | 7.4 | -- | EN | B1ab(i,ii,iii)+2ab(i,ii,iii) |
| *B. tumburanoensis* | 3 | 0 | 2 | 2 | 8* | 8 | 2 | 6.3 | 4 | EN | B1ab(iii)+2ab(iii) |
| *B. varipeltata* | 5 | 0 | 5 | 5 | 16* | 16 | 1 | 12 | -- | CR | B1ab(i,ii,iii) |
| *B. vermeulenii* | 1 | 2 | 3 | 3 | 12* | 12 | 2 | 8.8 | 4 | EN | B1ab(i,ii,iii)+2ab(i,ii,iii) |
| *B. watuwilensis* | 8 | 2 | 8 | 8 | 4112 | 28 | 4 | 3682 | 1082 | EN | B1ab(i,ii,iii)+2ab(i,ii,iii) |
| *B. willemii* | 12 | 0 | 10 | 9 | 23442 | 32 | 6 | 5184 | 428 | VU | B2ab(i,ii,iii) |

* Set to AOO estimate following the IUCN guidelines: "If EOO is less than AOO, EOO should be changed to make it equal to AOO to ensure consistency with the definition of AOO as an area within EOO."[3]

**Supplementary Information Table 2.** Endemicity, elevational range and forest cover loss.

| Species | **Sulawesi endemic** [Y=yes; N=no] | **Limestone endemic** [Y=yes; N=no] | **Elevational range** [m] | **Elevational range** [range category[15]] | **Tree cover loss 2001-2022** [ha] | **Tree cover loss 2001-2022** [% total] | **Tree cover loss due to fire 2001-2022** [ha] |
|---|---|---|---|---|---|---|---|
| *B. aptera* | N | N | 0-2583 | lowland to tropalpine | 172000 | 9 | 152000 |
| *B. balgooyi* | Y | Y | 72-1246 | lowland to upland | 9240 | 3.5 | 422 |
| *B. batusangiensis* | Y | Y | 959-1250 | upland | 138 | 18 | 4 |
| *B. bonthainensis* | Y | N | 1550-2134 | montane | 467 | 7.2 | 213 |
| *B. capituliformis* | Y | N | 500-1178 | hill to upland | 5720 | 4.9 | 451 |
| *B. carnosa* | Y | N | 5-370 | lowland | 29 | 4.4 | 0.46 |
| *B. celebica* | Y | N | 1050-1868 | upland to montane | 3800 | 4.5 | 62 |
| *B. chiasmogyna* | Y | N | 315-800 | lowland to hill | 9780 | 7.4 | 227 |
| *B. comestibilis* | Y | N | 34-2583 | lowland to tropalpine | 125000 | 7 | 8500 |
| *B. cuneatifolia* | Y | N | 300-1000 | lowland to upland | 105000 | 10 | 2720 |
| *B. didyma* | Y | N | 1050-1908 | upland to montane | 157 | 2.3 | 0.6 |
| *B. enoplocampa* | Y | N | 38-550 | Lowland to hill | 16 | 4.4 | 0.2 |
| *B. flacca* | Y | Y | 0-257 | lowland | 198000 | 15 | 6270 |
| *B. gambutensis* | Y | N | 700-1150 | hill to upland | 78 | 10 | 2 |
| *B. gemella* | Y | Y | 19-280 | lowland | 93 | 24 | 2 |
| *B. guttapila* | Y | N | 1359 | upland | 125 | 44 | 2 |
| *B. hekensis* | Y | N | 870-1120 | upland | 35 | 3 | 6 |
| *B. hispidissima* | Y | N | 1000-1404 | upland | 109 | 11 | 12 |
| *B. hooveriana* | Y | Y | 200-1618 | lowland to montane | 58200 | 6.4 | 5710 |
| *B. humilicaulis* | Y | unknown | unknown | unknown | -- | -- | -- |
| *B. ignita* | Y | unknown | unknown | unknown | -- | -- | -- |
| *B. imperfecta* | Y | N | 1400 | upland | -- | -- | -- |
| *B. incudiformicarpa* | Y | Y | 5-180 | lowland | 116 | 16 | 2 |
| *B. insueta* | Y | N | 100 | lowland | 6 | 2.9 | 0.1 |
| *B. insularum* | Y | N | 203-800 | lowland to hill | 18 | 1.4 | 0.1 |
| *B. iskandariana* | Y | Y | 0-200 | lowland | 421 | 8.6 | 11 |
| *B. johntanii* | Y | N | 40 | lowland | 185 | 48 | 20 |
| *B. kabaenensis* | Y | Y | 1000-1250 | upland | 72 | 18 | 3 |
| *B. kinhoi* | Y | N | 342-1200 | lowland to upland | 300 | 4.4 | 8 |
| *B. labengkiensis* | Y | Y | 1 | lowland | 1.1 | 0.4 | 0.7 |
| *B. lasioura* | Y | N | 474-1000 | hill to upland | 184 | 5 | 4 |
| *B. mabberleyana* | Y | Y | 70-490 | lowland to hill | 534 | 18 | 5 |
| *B. macintyreana* | Y | N | 130-182 | lowland | 60 | 16 | 2 |

| | | | | | | | |
|---|---|---|---|---|---|---|---|
| *B. masarangensis* | Y | N | 1050-1800 | upland to montane | 22 | 2.5 | 0.1 |
| *B. matarombeoensis* | Y | Y | 2-204 | lowland | 1290 | 4.3 | 11 |
| *B. mattampensis* | Y | Y | 5-10 | lowland | 1 | 1.4 | 0 |
| *B. medicinalis* | Y | N | 350-700 | lowland to upland | 41 | 5.8 | 2 |
| *B. mekonggensis* | Y | N | 122-1535 | lowland to montane | 62500 | 15 | 3380 |
| *B. mendumiae* | Y | N | 18-556 | lowland to hill | 376000 | 13 | 51600 |
| *B. nobmanniae* | Y | N | 879-1050 | upland | 31 | 2.7 | 0 |
| *B. ozotothrix* | Y | N | 200-871 | lowland to upland | 551000 | 14 | 37600 |
| *B. pitopangii* | Y | N | 20-250 | lowland | 73 | 18 | 0.1 |
| *B. prionota* | Y | Y | 1013-2182 | upland to montane | 346 | 7.8 | 5 |
| *B. rachmatii* | Y | N | 881-1442 | upland | 4530 | 8.9 | 401 |
| *B. ranoposoensis* | Y | Y | 100-1784 | upland to montane | 18500 | 13 | 1010 |
| *B. rantemarioensis* | Y | N | 1496-1978 | upland to montane | 164 | 4.9 | 7 |
| *B. rieckei* | N | N | 10-2339 | lowland to montane | 4170000 | 5.1 | 239000 |
| *B. robusta* | N | N | 800-2572 | upland to tropalpine | 1110000 | 14 | 68900 |
| *B. rolandfadlii* | Y | N | 280 | lowland | 27 | 6.9 | 0.5 |
| *B. sanguineopilosa* | Y | N | 1824-2070 | montane | 54 | 12 | 13 |
| *B. sarasinorum* | Y | N | 571-764 | hill | 130 | 16 | 19 |
| *B. siccacaudata* | Y | Y | 20-299 | lowland | 1890 | 3.3 | 99 |
| *B. sidolensis* | Y | N | 800-1000 | hill to upland | 1 | 0.3 | 0 |
| *B. siregarii* | Y | Y | 811-855 | hill to upland | 35 | 7.3 | 0 |
| *B. sojolensis* | Y | N | 166-600 | lowland to hill | 1010 | 6.6 | 13 |
| *B. sphenocarpa* | Y | N | 871-1326 | upland | 41 | 5.2 | 3 |
| *B. stevei* | Y | N | 660 | hill | 11 | 2.7 | 3 |
| *B. strachwitzii* | Y | N | 192-760 | lowland to hill | 821 | 7.6 | 42 |
| *B. tjiasmantoi* | Y | N | 900-1100 | upland | 67 | 18 | 14 |
| *B. torajana* | Y | N | 800-1970 | hill to montane | 64 | 3.1 | 7 |
| *B. tumburanoensis* | Y | Y | 109-302 | lowland | 91 | 12 | 0.1 |
| *B. varipeltata* | Y | N | 92-1080 | lowland to upland | 369 | 27 | 23 |
| *B. vermeulenii* | Y | Y | 1126-1700 | upland to montane | 144 | 13 | 40 |
| *B. watuwilensis* | Y | Y | 115-1200 | lowland to upland | 9080 | 2.3 | 150 |
| *B. willemii* | Y | Y | 10-482 | lowland to hill | 113000 | 12 | 4220 |

**Supplementary Information Table 3.** Habitat descriptions.

| Species | Habitat |
| --- | --- |
| *B. aptera* | The species has a relatively wide habitat tolerance. It grows terrestrially in lowland to montane forest from sea-level to ca. 2600 m elevation, in primary and secondary forest vegetation, and severely disturbed forest and forest margins at roadsides and plantations. |
| *B. balgooyi* | This limestone-endemic species grows terrestrially in lowland to upland forest at ca. 70 to 1250 m elevation, in primary or secondary forest vegetation, on steep slopes and river embankments, in humid and shaded places. |
| *B. batusangiensis* | This limestone-endemic species grows lithophytically in upland limestone karst habitats at ca. 950 to 1250 m elevation, in rock crevices on vertical limestone cliffs, in open areas exposed to direct sunlight. |
| *B. bonthainensis* | This species grows terrestrially in montane forest at about 1550 to 2350 m elevation, in primary and secondary forest vegetation, often on stream embankments, in dense shade. Locally abundant. |
| *B. capituliformis* | This species grows terrestrially in hill and upland forest at 500 to 1200 m elevation, in primary and secondary forest. |
| *B. carnosa* | This species grows terrestrially in lowland forest from about five to 370 m elevation. The only known recent collection of this species is from strongly disturbed vegetation in a village garden, in full shade. |
| *B. celebica* | This species grows terrestrially in upland to montane forest at about 1050 to 1900 m elevation, on the forest floor or on mossy rocks at stream embankments, in full shade to open areas. |
| *B. chiasmogyna* | This species grows terrestrially in lowland to hill forest at about 300 to 800 m elevation, in primary forest vegetation, in stream- or riverside habitats, in full shade. |
| *B. comestibilis* | This species grows terrestrially in lowland to montane forest at ca. 30 to 2500 m elevation, in primary to strongly disturbed forest vegetation, at road sides and margins of coffee plantations, in full to half-shade, sometimes in open, sun-exposed habitats, but then plants with signs of sun damage. |
| *B. cuneatifolia* | This species grows terrestrially in lowland to upland forest at ca. 300 to 1000 m elevation, in primary to secondary forest vegetation. |
| *B. didyma* | This species grows terrestrially in upland to montane forest from ca. 1050 to 1910 m elevation, in primary to disturbed forest vegetation, on rocky ground. |
| *B. enoplocampa* | This species grows terrestrially or lithophytically in lowland to hill forest at ca. 40 to 550 m elevation, in primary to strongly disturbed forest vegetation, on river embankments and in village gardens, in soil or in between and on large volcanic rocks. |
| *B. flacca* | This limestone-endemic species grows lithophytically in lowland forest from sea-level to ca. 260 m elevation, in primary to disturbed forest vegetation, on vertical limestone cliffs, limestone boulders, steep slopes, cave mouths, and shaded road embankments. |
| *B. gambutensis* | This species grows terrestrially in hill to upland rainforest at ca. 700 to 1150 m elevation, in primary forest vegetation, in river-side habitats. |

| | |
|---|---|
| *B. gemella* | This limestone-endemic species grows lithophytically in limestone karst habitats in lowland forest at ca. 20 to 280 m elevation, in secondary forest vegetation, on limestone walls, in half-shade to full shade. |
| *B. guttapila* | This species grows terrestrially in upland forest at ca. 1360 m elevation. |
| *B. hekensis* | This species grows terrestrially in upland forest at ca. 850 to 1150 m elevation, in primary forest vegetation, often along the sides of small streams. |
| *B. hispidissima* | This species grows terrestrially in upland forest at ca. 1000 to 1400 m elevation, in secondary forest vegetation, reported from semi-shaded habitats including steep slopes and level, marshy terrain. |
| *B. hooveriana* | This species grows terrestrially or lithophytically in lowland to montane forests from sea-level to about 1600 m elevation, in secondary to severely disturbed forest vegetation, in semi-shade to open habitats, frequently on limestone but also recorded on granite. |
| *B. humilicaulis* | unknown |
| *B. ignita* | unknown |
| *B. imperfecta* | This species grows terrestrially in upland forest at ca. 1400 m elevation. |
| *B. incudiformicarpa* | This limestone-endemic species grows lithophytically in lowland forest from sea-level to about 180 m elevation, on limestone cliffs and coralline limestone rocks, in primary to strongly disturbed forest vegetation, in partial to full shade. |
| *B. insueta* | This species grows terrestrially in lowland forest at ca. 100 m elevation; it was observed growing on a rocky slope, in full shade. |
| *B. insularum* | This species grows terrestrially in hill forest at around 800 m elevation, in primary and secondary forest. |
| *B. iskandariana* | This limestone-endemic species grows lithophytically in lowland limestone karst habitats from sea-level to about 200 m elevation, including limestone cliff walls, cave mouths and stalactites at cave entrances. |
| *B. johntanii* | This species grows terrestrially in lowland forest at around 100 m elevation, in secondary forest vegetation; it was observed on slopes at the side of a small stream. |
| *B. kabaenensis* | This limestone-endemic species grows lithophytically in upland limestone karst habitats at ca. 1000 to 1250 m elevation, at the base of limestone cliffs, in humid and shaded areas. |
| *B. kinhoi* | This species grows terrestrially in lowland to upland forest at ca. 340 to 1200 m elevation, in secondary forest to strongly disturbed forest vegetation. |
| *B. labengkiensis* | This limestone-endemic species grows lithophytically in lowland limestone karst habitats, in crevices of vertical limestone cliffs in coastal limestone forest, in shaded places. |
| *B. lasioura* | This species grows lithophytically in hill to upland forest between ca. 450 and 1000 m elevation, in primary forest vegetation and primary forest margins, on rocks and rock walls, in full shade. |
| *B. mabberleyana* | This limestone-endemic species grows terrestrially or lithophytically in lowland to hill forest at ca. 50 to 500 m elevation, in primary to severely disturbed secondary forest vegetation, terrestrially on limestone-derived soils or lithophytically on limestone rock, often along small streams and steep slopes along rivers, in dense to partial shade. |
| *B. macintyreana* | This species grows terrestrially in lowland forest at ca. 100 to 200 m elevation, in secondary forest, on steep slopes, in full shade. |

| | |
|---|---|
| *B. masarangensis* | This species grows terrestrially in upland to montane forest at ca. 1000 to 1800 m elevation, in primary and secondary forest vegetation, in full shade. |
| *B. matarombeoensis* | This limestone-endemic species grows lithophytically in lowland forest in limestone karst areas from sea-level to about 200 m elevation, on vertical limestone walls, in half-shade to full-shade. During the dry season the species was reported to be dormant. |
| *B. mattampensis* | This limestone-endemic species grows lithophytically in lowland limestone karst habitats from sea-level to about 10 m elevation, creeping on vertical limestone walls, at cave entrances. |
| *B. medicinalis* | This species grows terrestrially or lithophytically in lowland to upland rainforest at ca. 350 to 700 m elevation, in primary forest vegetation, on or between larger rocks, in shady and humid places. |
| *B. mekonggensis* | This species grows terrestrially in lowland to montane rainforest at ca. 100 to 1550 m elevation, in primary and secondary forest, disturbed forest margins and strongly disturbed vegetation on road embankments, frequently observed on limestone-derived soils, in full shade to partial shade. |
| *B. mendumiae* | The species grows terrestrially or lithophytically in lowland to hill forest from sea-level to about 560 m elevation, in secondary forest to strongly disturbed vegetation, frequently on limestone rocks or limestone-derived soil, but also observed on other substrates, in full to partial shade. |
| *B. nobmanniae* | This species was observed growing lithophytically at the margin of upland forest at ca. 850 to 1000 m elevation, at the margin of a secondary forest patch, on ledges and in crevices on an almost vertical rock wall. |
| *B. ozotothrix* | This species grows terrestrially or lithophytically in lowland to upland forest from sea-level to ca. 900 m elevation, in primary, secondary or severely disturbed forest vegetation, either lithophytically on limestone rock or mossy granite, or terrestrially in soil on limestone bedrock or alluvial soils, in full shade to partial shade. |
| *B. pitopangii* | This species grows terrestrially in lowland forest from sea-level to about 250 m elevation, in secondary forest vegetation, in full shade. |
| *B. prionota* | This limestone-endemic species grows terrestrially or lithophytically in limestone karst habitats in upland to montane rainforest between ca. 1000 and 2200 m elevation, in primary, secondary and strongly disturbed forest vegetation, lithophytically on limestone rock walls or terrestrially on steep slopes or river banks on limestone-derived soils, in full shade to open places. |
| *B. rachmatii* | This species grows terrestrially in upland forest between ca. 880 and 1450 m elevation, in primary and secondary forest and strongly disturbed forest patch remnants, at stream margins and on slopes lining rivers. |
| *B. ranoposoensis* | This limestone-endemic species grows lithophytically or terrestrially in lowland to montane forest at ca. 100 to 1800 m elevation, in primary and secondary forest, lithophytically on vertical limestone cliffs, cave entrances and limestone rocks, or terrestrially on soil on limestone bedrock at stream margins, on slopes and on road embankments, in full shade. |
| *B. rantemarioensis* | This species grows terrestrially in upland to montane rainforest at ca. 1500 to 2000 m elevation, in primary and secondary rainforest vegetation, on steep slopes and ridges, in humid places, in full to partial shade. Locally abundant. |
| *B. rieckei* | This species grows terrestrially in lowland to montane forests from sea-level to ca. 2350 m elevation, in primary forest, secondary forest, and severely disturbed forest vegetation, in full to partial shade. |

| | |
|---|---|
| *B. robusta* | This species grows terrestrially in hill to montane rainforest at ca. 800 to 2600 m elevation. |
| *B. rolandfadlii* | This species grows terrestrially in lowland forest, on steep slopes with thick leaf litter layer, in dense shade. |
| *B. sanguineopilosa* | This species grows terrestrially in montane forest at ca. 1800 to 2100 m elevation, in primary forest and secondary forest, on steep slopes. |
| *B. sarasinorum* | This species grows terrestrially in hill forest at ca. 550 to 800 m elevation, in primary and secondary forest, on slopes and road embankments, in full shade to half-shade. |
| *B. siccacaudata* | This limestone-endemic species grows lithophytically in lowland limestone karst habitats from sea-level to about 300 m elevation, in primary to strongly disturbed lowland forest on limestone, on the limestone cliff walls, cave entrances and on limestone boulders, in partial to full shade. |
| *B. sidolensis* | This species grows terrestrially in hill to upland forest at ca. 800 to 1000 m elevation, on steep slopes, in dense shade. |
| *B. siregarii* | This limestone-endemic species grows terrestrially or lithophytically in hill to upland limestone karst habitats at ca. 800 to 860 m elevation, in secondary forest and strongly disturbed forest vegetation, lithophytically on limestone cliffs and limestone outcrops, terrestrially at the base of limestone cliffs, on road embankments, in full shade to open places. |
| *B. sojolensis* | This species grows terrestrially in lowland to hill forest at ca. 150 to 600 m elevation, in secondary forest, in disturbed habitats, on steep, rocky river embankments and rocky slopes, in full shade. |
| *B. sphenocarpa* | This species grows terrestrially in upland forest at ca. 850 to 1350 m elevation, secondary forest, disturbed forest margins, road embankments, in full shade. |
| *B. stevei* | This species grows terrestrially in hill forest. |
| *B. strachwitzii* | This species grows terrestrially in lowland to hill forest at ca. 190 to 800 m elevation, in secondary forest. It has been reported from humus-covered slopes, damp and flat places, and disturbed riverside vegetation, in full shade. |
| *B. tjiasmantoi* | This species grows terrestrially in upland forest at ca. 900 to 1100 m elevation, in strongly disturbed secondary forest, on steep slopes of an irrigation water channel in a coffee plantation, in dense shade. |
| *B. torajana* | This species grows terrestrially in hill to montane forest between ca. 800 and 2000 m elevation, in primary and secondary forest and strongly disturbed forest remnants, in strongly disturbed habitats at forest margins, on steep slopes and level ground, besides small springs and streams, on road embankments, usually in humid places, in full shade to partial shade. |
| *B. tumburanoensis* | This limestone-endemic species grows lithophytically in lowland limestone karst forest habitats at ca. 100 to 300 m elevation, on limestone walls and limestone boulders on steep slopes at waterfalls. |
| *B. varipeltata* | This species grows terrestrially or lithophytically in lowland to upland forest at ca. 90 to 1100 m elevation, in primary and secondary forest, lithophytically on wet, riverside rock walls or terrestrially in thickets. |
| *B. vermeulenii* | This limestone-endemic species grows terrestrially in upland to montane limestone forest at ca. 1100 to 1700 m elevation, in remnant primary forest pockets on limestone slopes. |

| | |
|---|---|
| *B. watuwilensis* | This limestone-endemic species grows terrestrially or lithophytically in lowland to upland limestone karst forest at ca. 100 to 1200 m elevation, in primary forest, on limestone cliffs and rocks, on moist stalactites, or terrestrially on steep slopes on limestone bedrock, in full shade to partial shade. |
| *B. willemii* | This limestone-endemic species grows lithophytically or terrestrially in lowland to hill limestone karst forest from sea-level to ca. 500 m elevation, in primary and secondary forests, in strongly disturbed forest vegetation, lithophytically on vertical cliffs or limestone boulders or terrestrially in soil at the base of limestone boulders, in full to partial shade. Locally abundant. |

**Supplementary Information Table 4.** Presence in legally protected areas.

| **Species** | **Protected area** |
|---|---|
| *B. aptera* | Bogani Nani Wartabone National Park (IUCN category II), Ganda Dewata National Park (IUCN category II), Lore Lindu National Park (IUCN category II) |
| *B. balgooyi* | Mangolo Nature Recreation Park (IUCN category V) |
| *B. batusangiensis* | |
| *B. bonthainensis* | Malino Nature Recreation Park (IUCN category V) |
| *B. capituliformis* | Gunung Ambang Nature Reserve (IUCN category III) |
| *B. carnosa* | |
| *B. celebica* | Lore Lindu National Park (IUCN category II) |
| *B. chiasmogyna* | Bogani Nani Wartabone National Park (IUCN category II), Nantu Wildlife Reserve (IUCN category III) |
| *B. comestibilis* | Ganda Dewata National Park (IUCN category II) |
| *B. cuneatifolia* | Nantu Wildlife Reserve (IUCN category III) |
| *B. didyma* | |
| *B. enoplocampa* | |
| *B. flacca* | Rawa Aopa Watumohai National Park (IUCN Category II), Tanjung Peropa Wildlife Reserve (IUCN category IV), Murhum (Nipa-Nipa) Grand Forest Park (IUCN category VI) |
| *B. gambutensis* | Bogani Nani Wartabone National Park (IUCN category II) |
| *B. gemella* | |
| *B. guttapila* | |
| *B. hekensis* | |
| *B. hispidissima* | Gunung Ambang Nature Reserve (IUCN category III) |
| *B. hooveriana* | Lore Lindu National Park (IUCN category II), Wera Nature Recreation Park (IUCN category V) |
| *B. humilicaulis* | |
| *B. ignita* | |
| *B. imperfecta* | |
| *B. incudiformicarpa* | |
| *B. insueta* | |
| *B. insularum* | |
| *B. iskandariana* | |
| *B. johntanii* | |
| *B. kabaenensis* | |
| *B. kinhoi* | |

| | |
|---|---|
| *B. labengkiensis* | |
| *B. lasioura* | Faruhumpenai Nature Reserve (IUCN category Ia) |
| *B. mabberleyana* | |
| *B. macintyreana* | Bogani Nani Wartabone National Park (IUCN category II) [at the border] |
| *B. masarangensis* | |
| *B. matarombeoensis* | |
| *B. mattampensis* | |
| *B. medicinalis* | |
| *B. mekonggensis* | Faruhumpenai Nature Reserve (IUCN category Ia), Banggai Kepulauan Nature Recreation Park (IUCN category V) |
| *B. mendumiae* | Pamona Nature Reserve (IUCN category Ia) |
| *B. nobmanniae* | |
| *B. ozotothrix* | Bantimurung Bulusaraung National Park (IUCN category II) |
| *B. pitopangii* | Gunung Sojol Nature Reserve (IUCN category Ia) |
| *B. prionota* | |
| *B. rachmatii* | |
| *B. ranoposoensis* | |
| *B. rantemarioensis* | |
| *B. rieckei* | Gunung Sojol Nature Reserve (IUCN category Ia), Pangi Binangga Nature Reserve (IUCN category Ia), Bogani Nani Wartabone National Park (IUCN category II), Lore Lindu National Park (IUCN category II), Gunung Duasaudara Nature Reserve (IUCN category III), Nantu Wildlife Reserve (IUCN category III) |
| *B. robusta* | |
| *B. rolandfadlii* | |
| *B. sanguineopilosa* | |
| *B. sarasinorum* | Lore Lindu National Park (IUCN category II) |
| *B. siccacaudata* | Bantimurung Bulusaraung National Park (IUCN category II) |
| *B. sidolensis* | |
| *B. siregarii* | |
| *B. sojolensis* | Gunung Sojol Nature Reserve (IUCN category Ia) |
| *B. sphenocarpa* | |
| *B. stevei* | |
| *B. strachwitzii* | Bogani Nani Wartabone National Park (IUCN category II) |
| *B. tjiasmantoi* | |
| *B. torajana* | |

| | |
|---|---|
| *B. tumburanoensis* | |
| *B. varipeltata* | |
| *B. vermeulenii* | |
| *B. watuwilensis* | |
| *B. willemii* | Nantu Wildlife Reserve (IUCN category III), Lombuyan I and II Wildlife Reserve (IUCN category IV) |

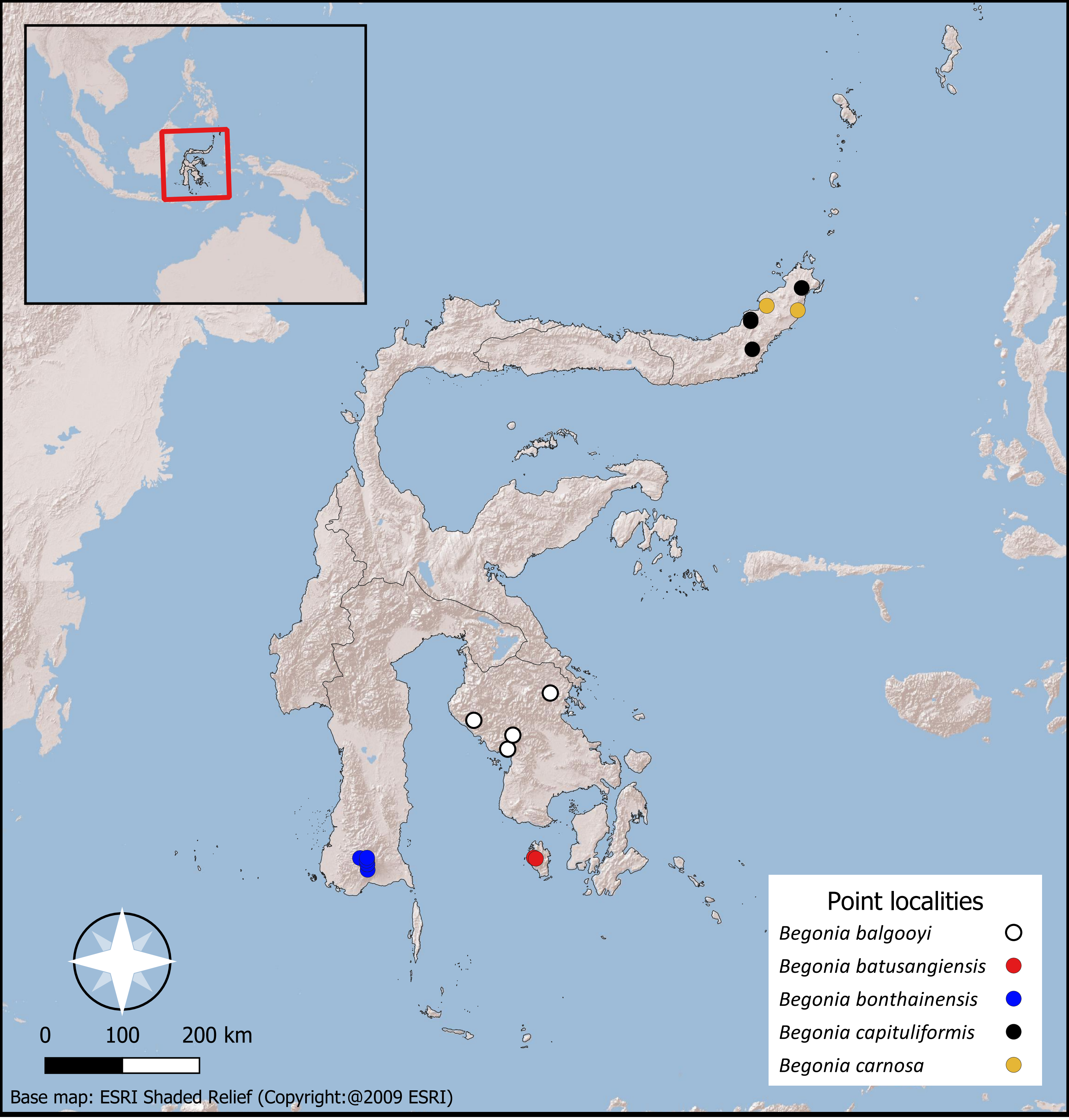


Supplementary Figure 1. Distribution map.

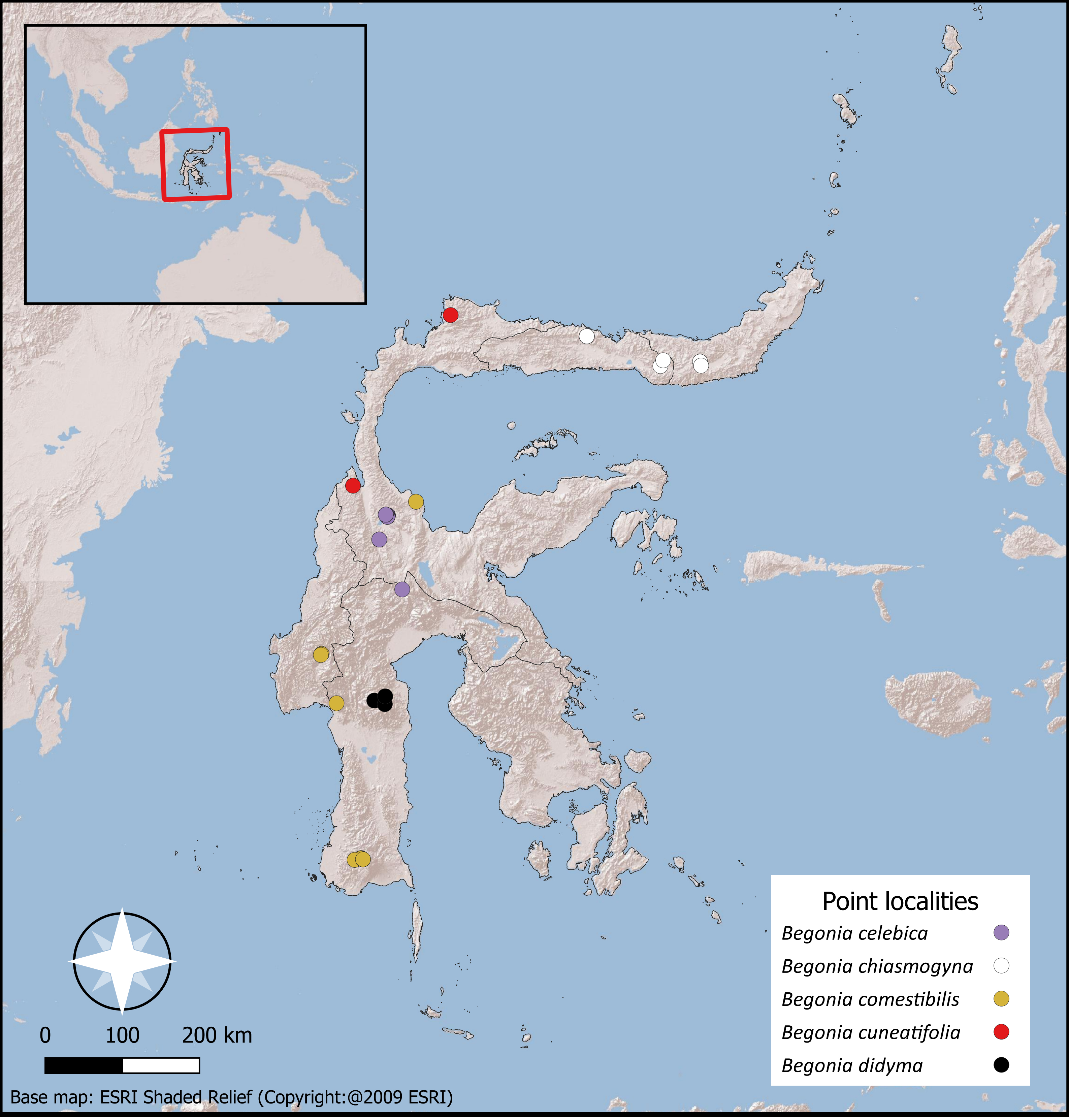


Supplementary Figure 2. Distribution map.

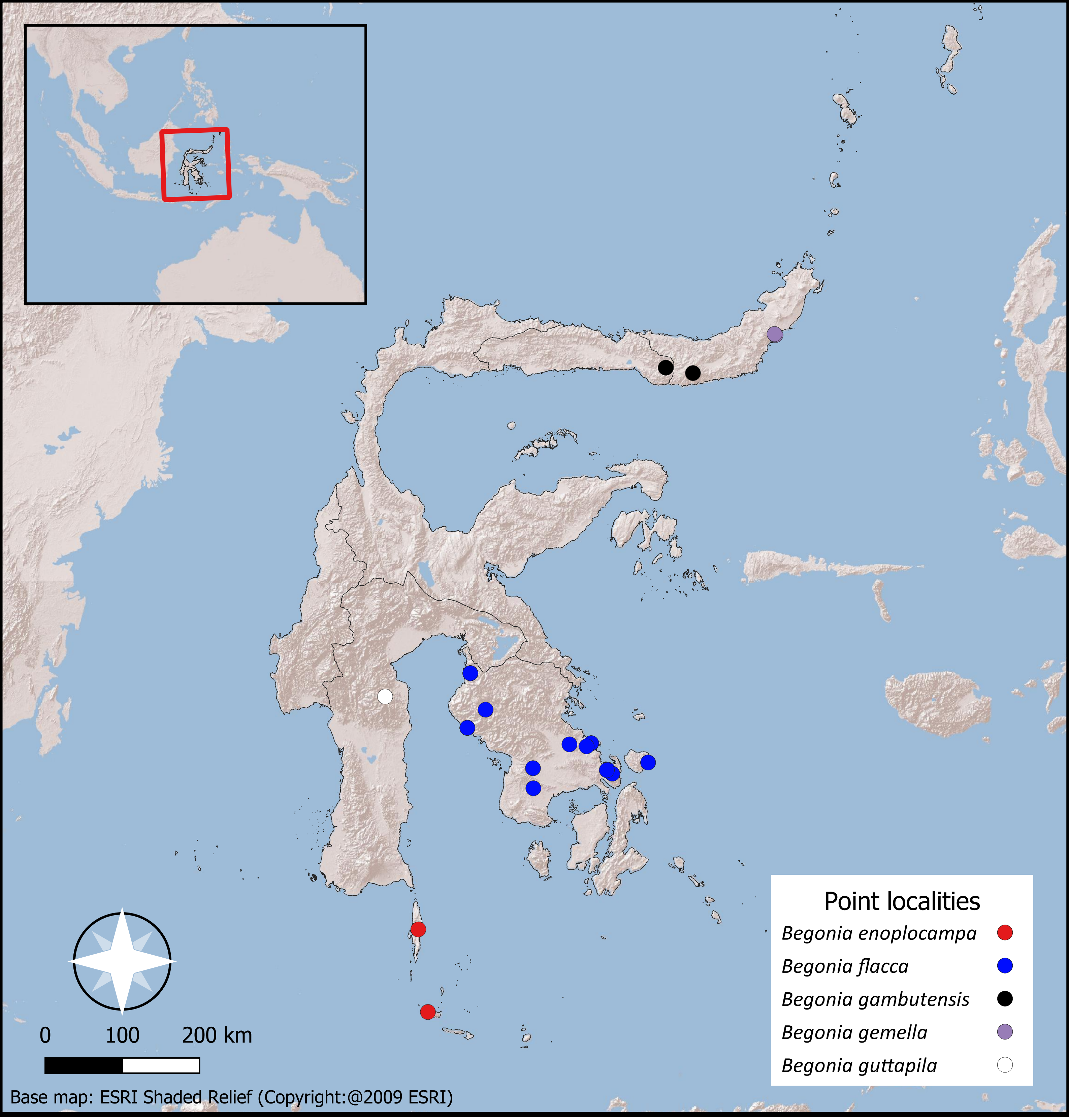


Supplementary Figure 3. Distribution map.

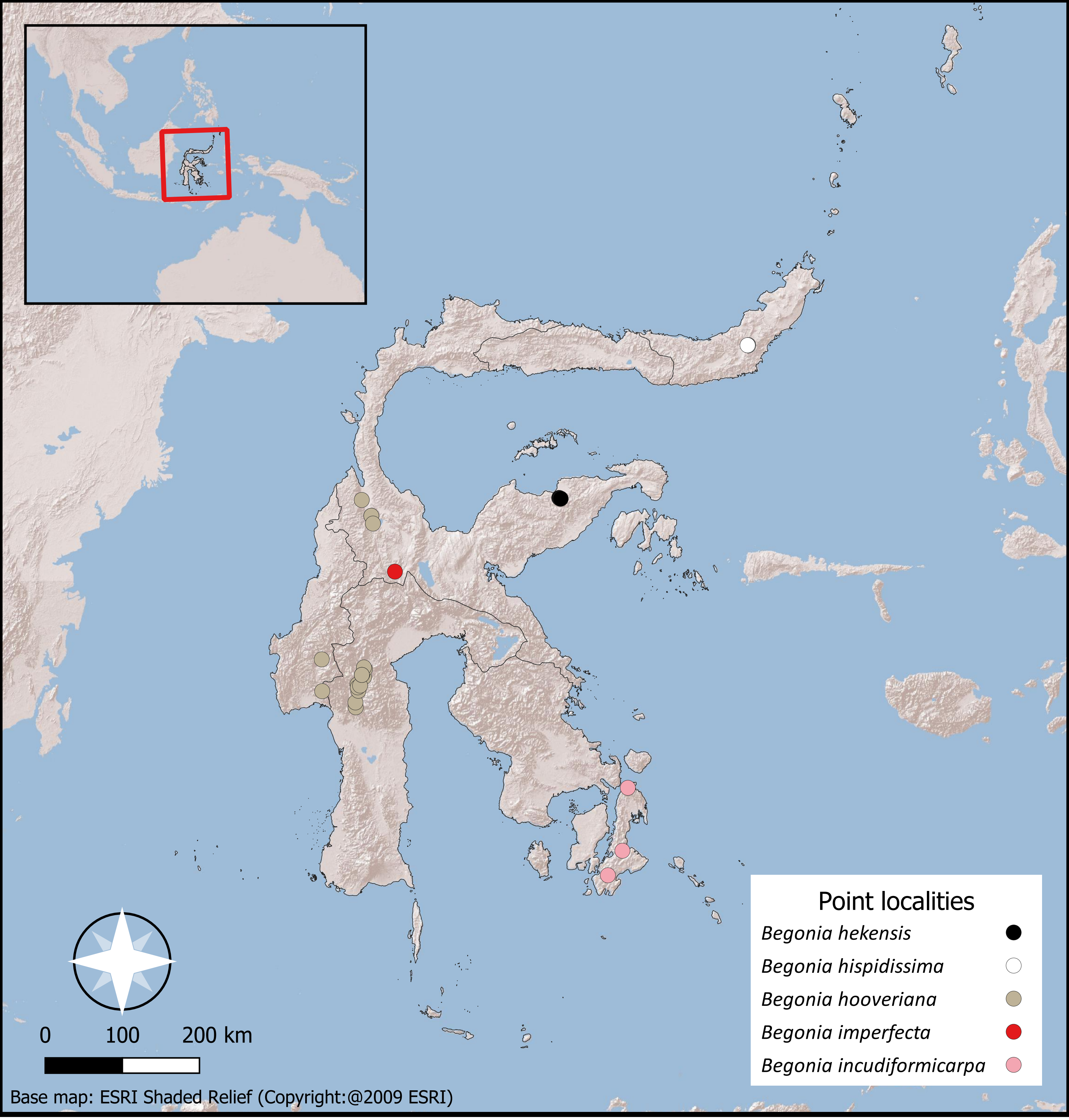


Supplementary Figure 4. Distribution map.

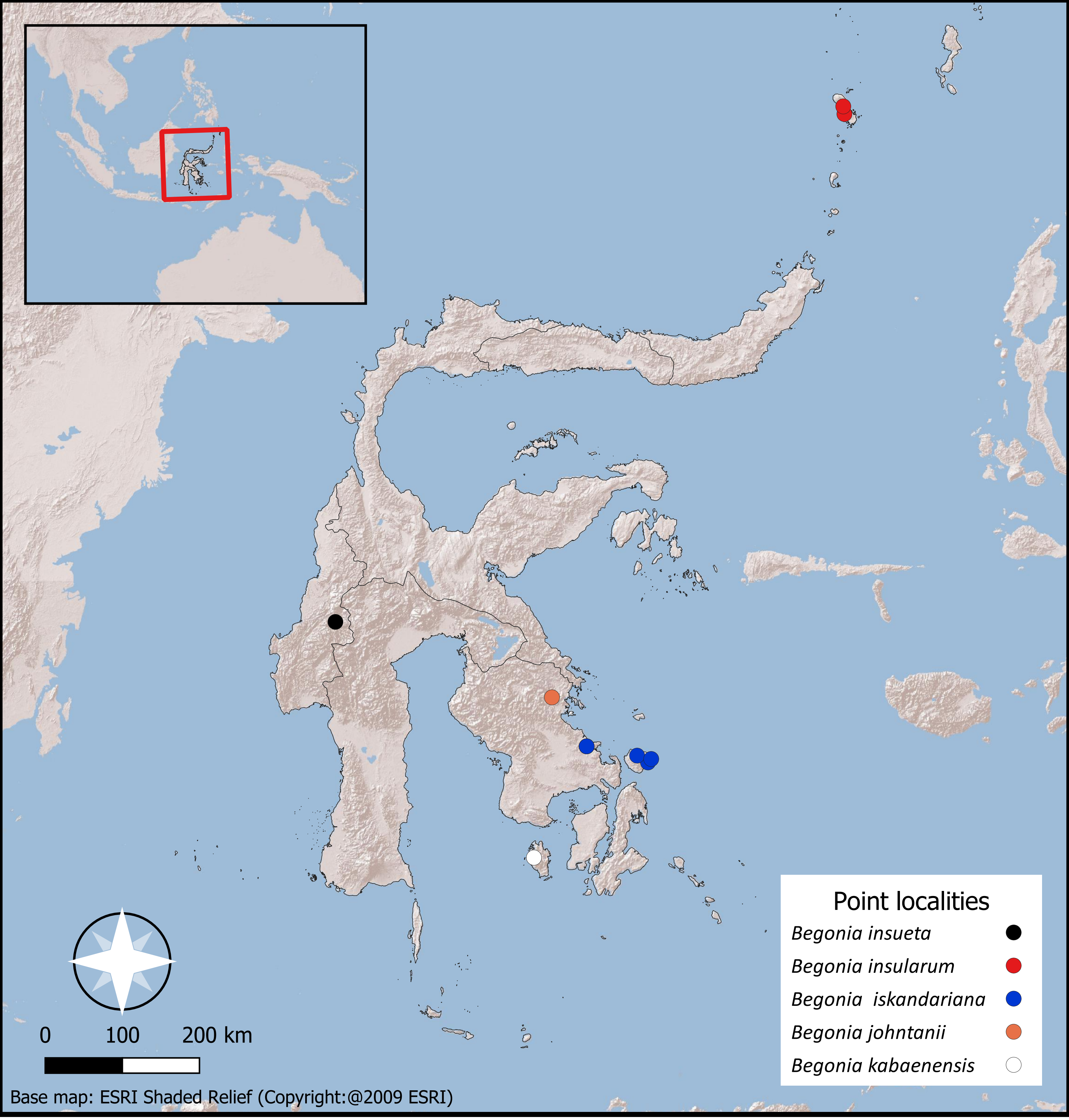


Supplementary Figure 5. Distribution map.

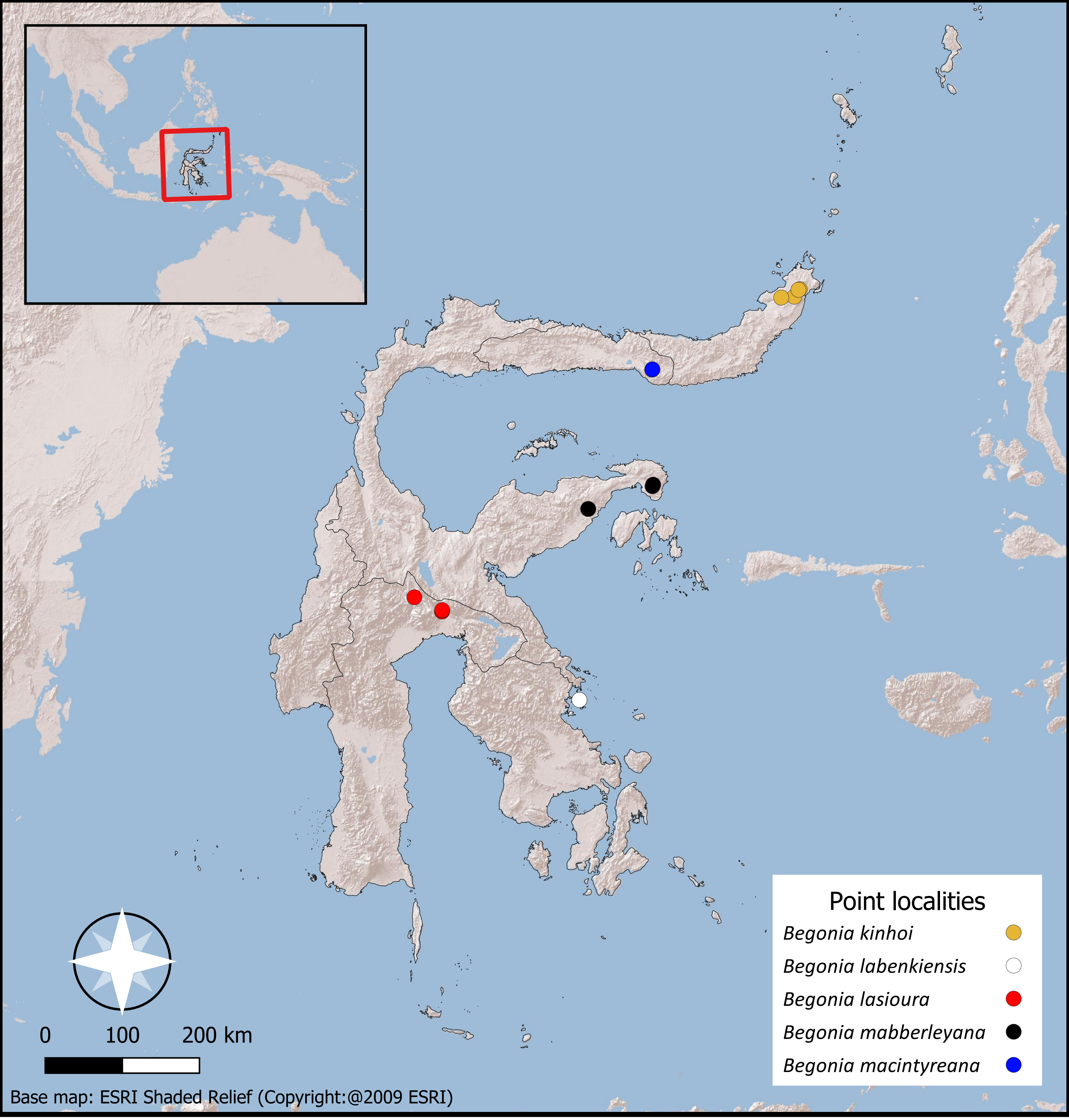


Supplementary Figure 6. Distribution map.

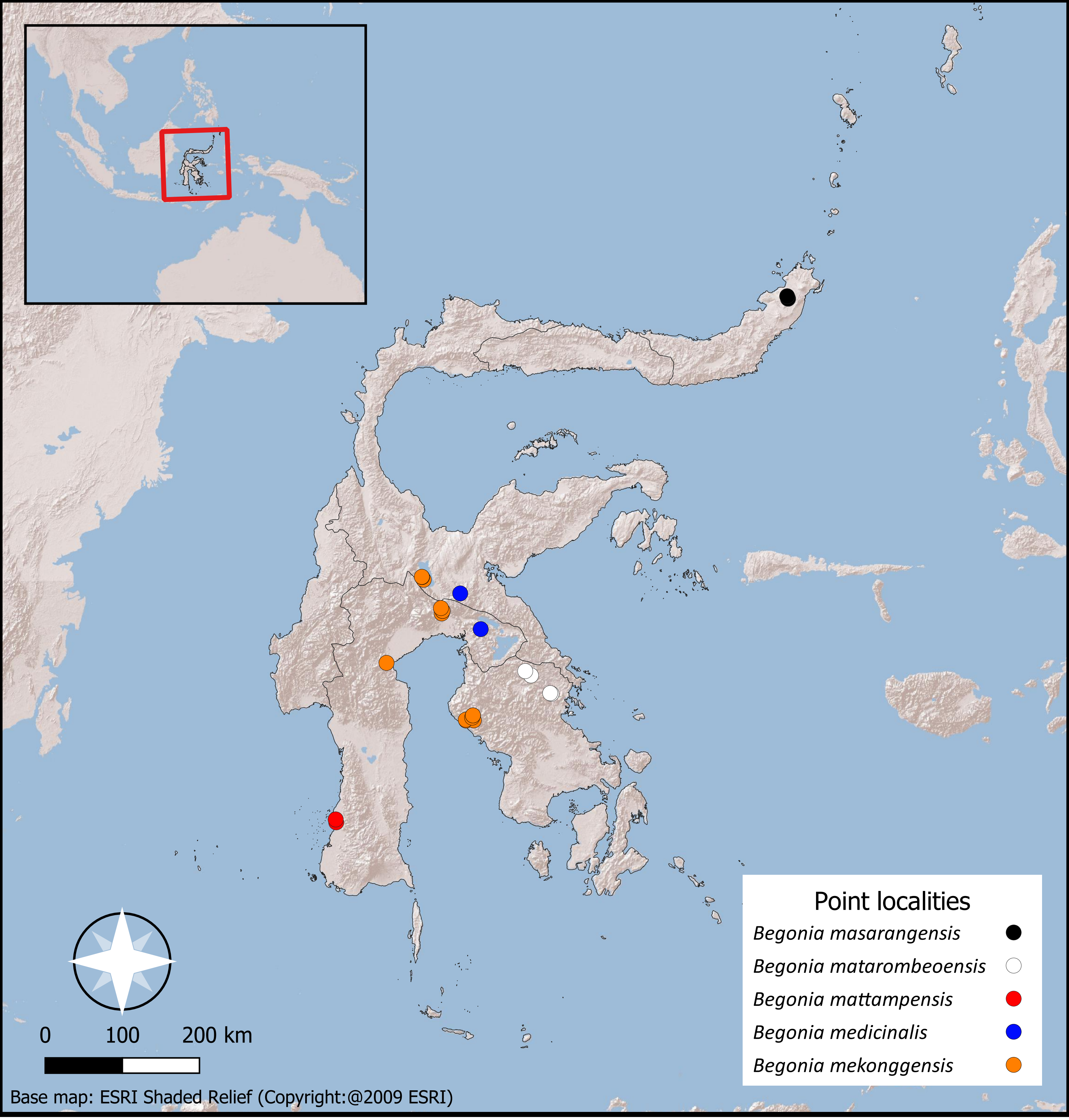


Supplementary Figure 7. Distribution map.

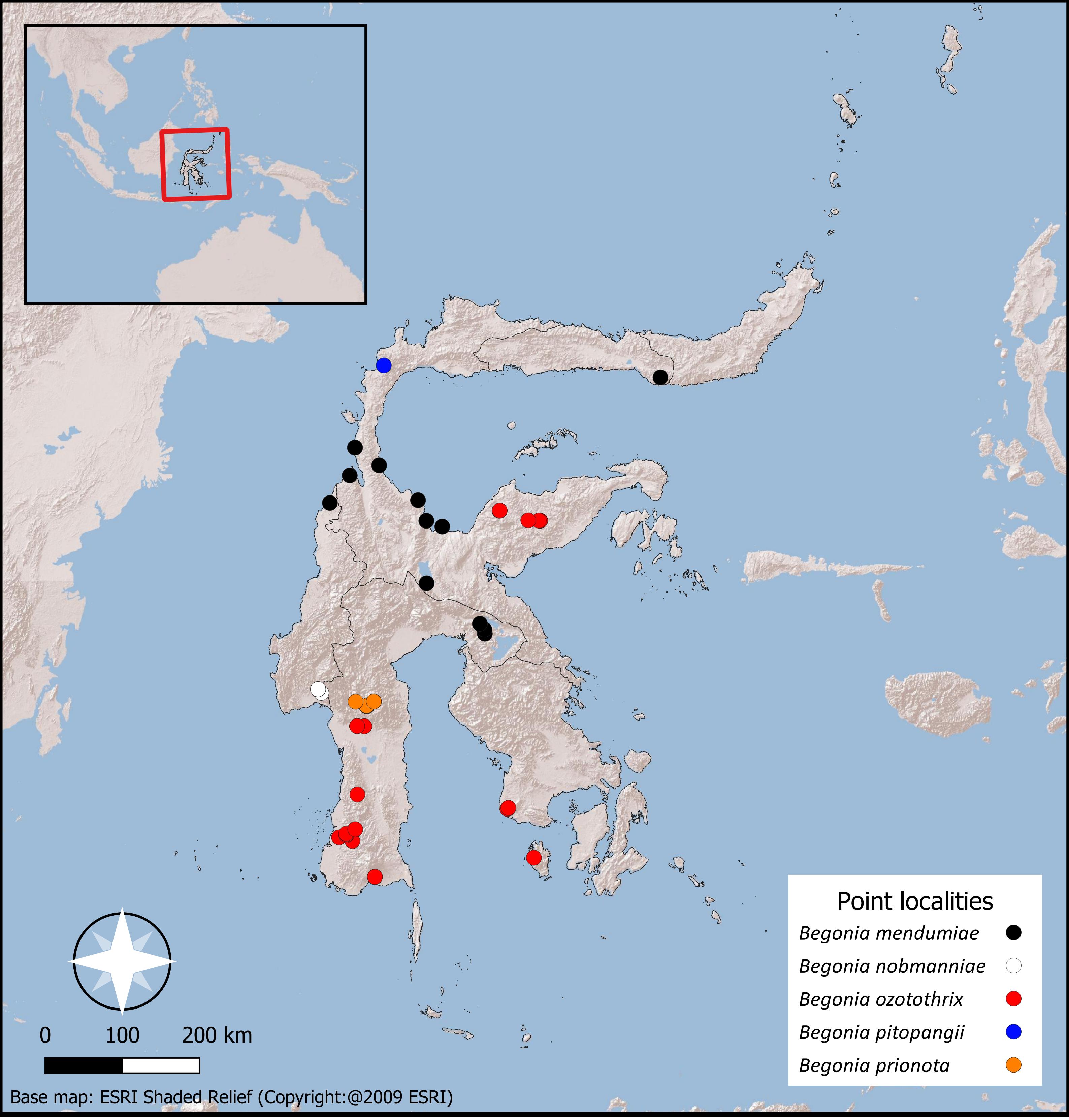


Supplementary Figure 8. Distribution map.

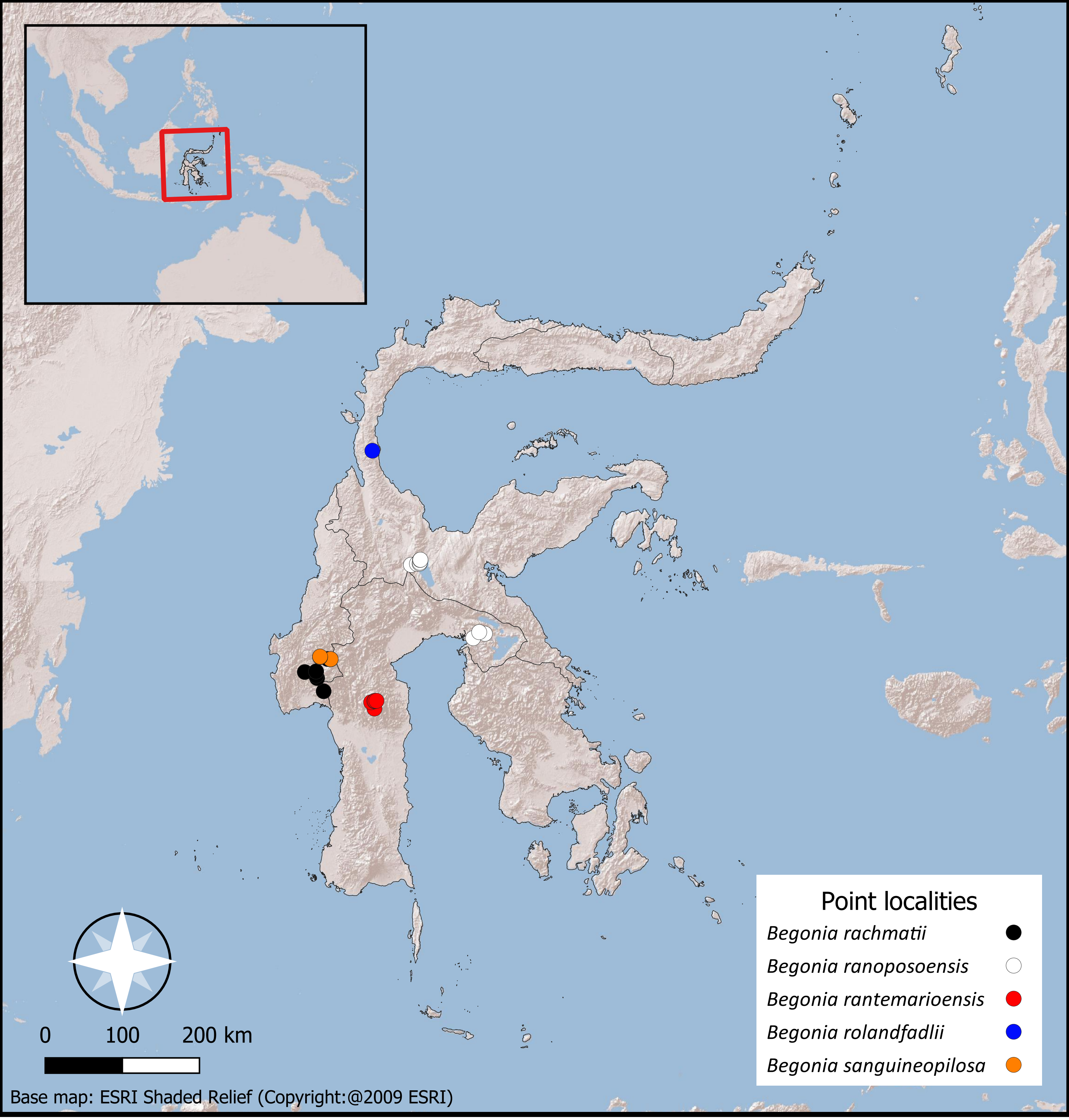


Supplementary Figure 9. Distribution map.

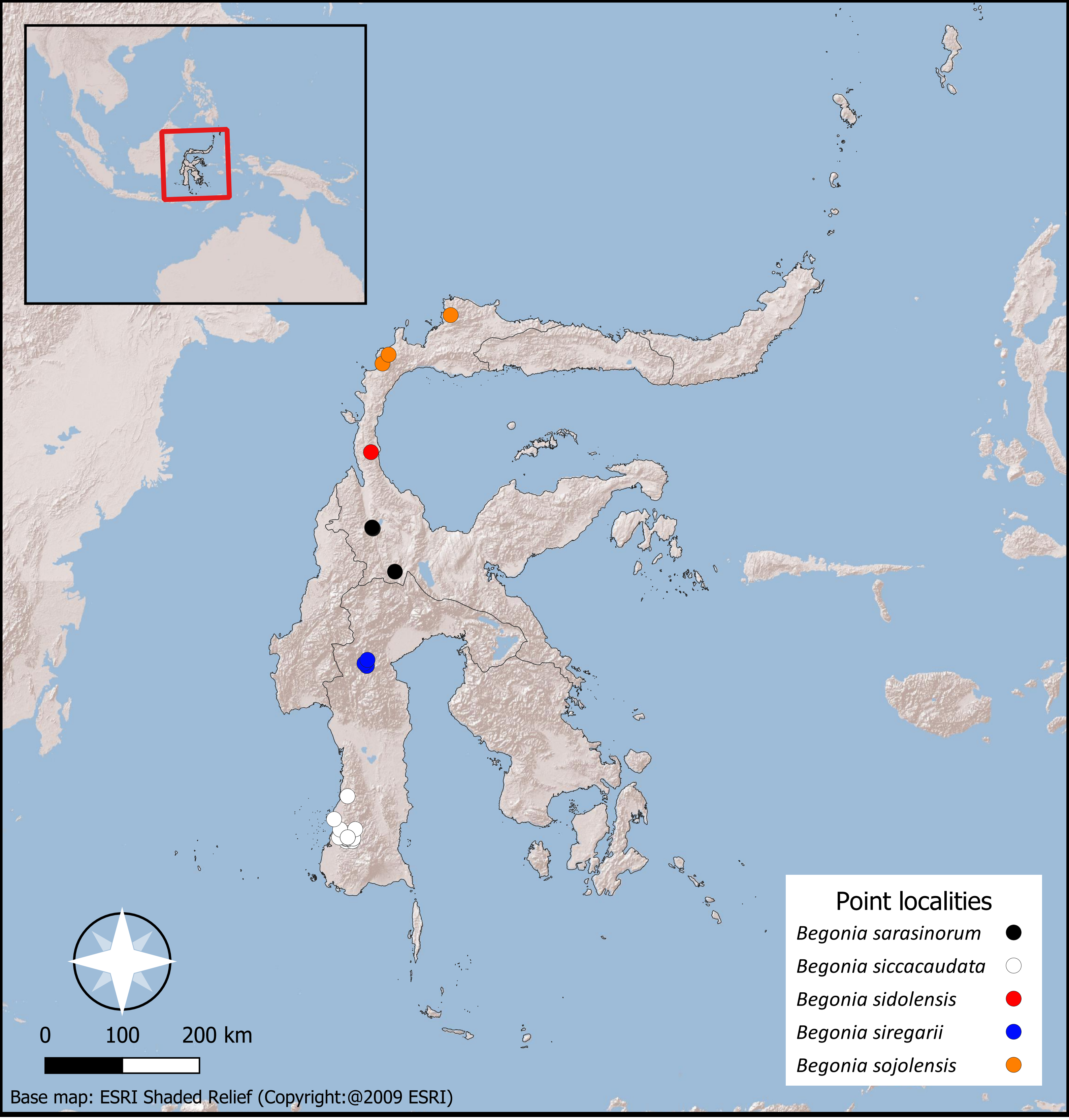


Supplementary Figure 10. Distribution map.

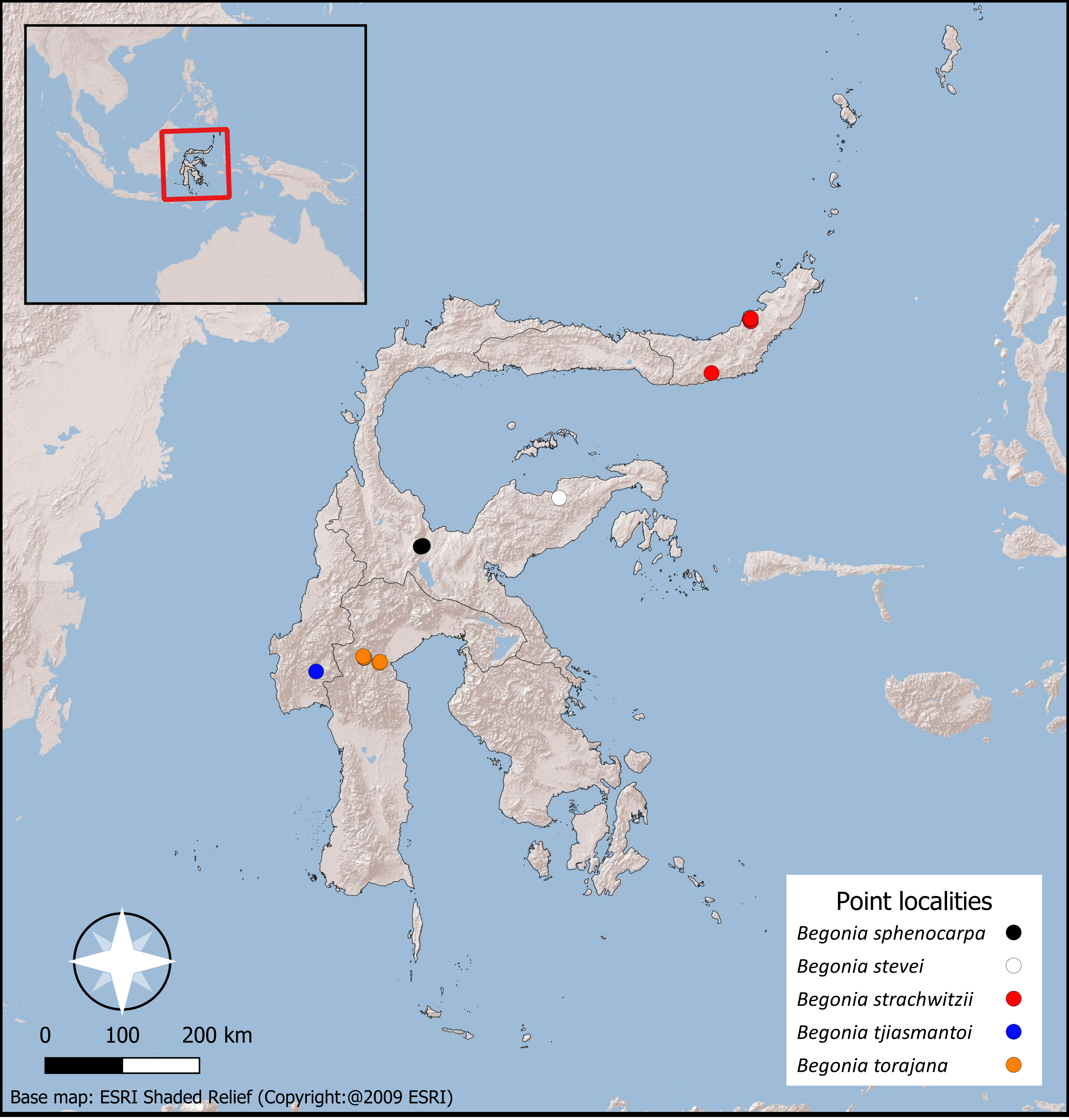


Supplementary Figure 11. Distribution map.

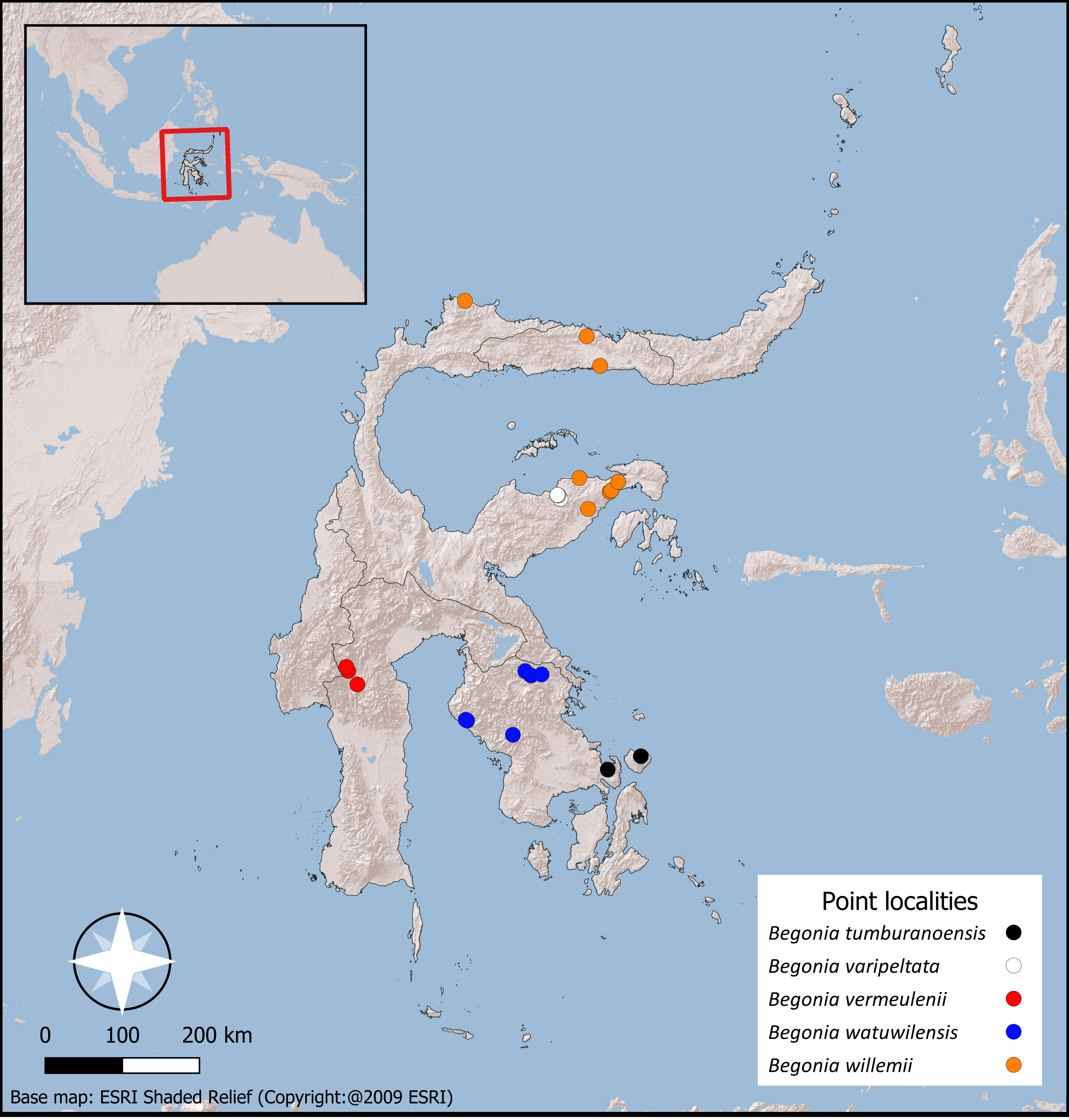


Supplementary Figure 12. Distribution map.

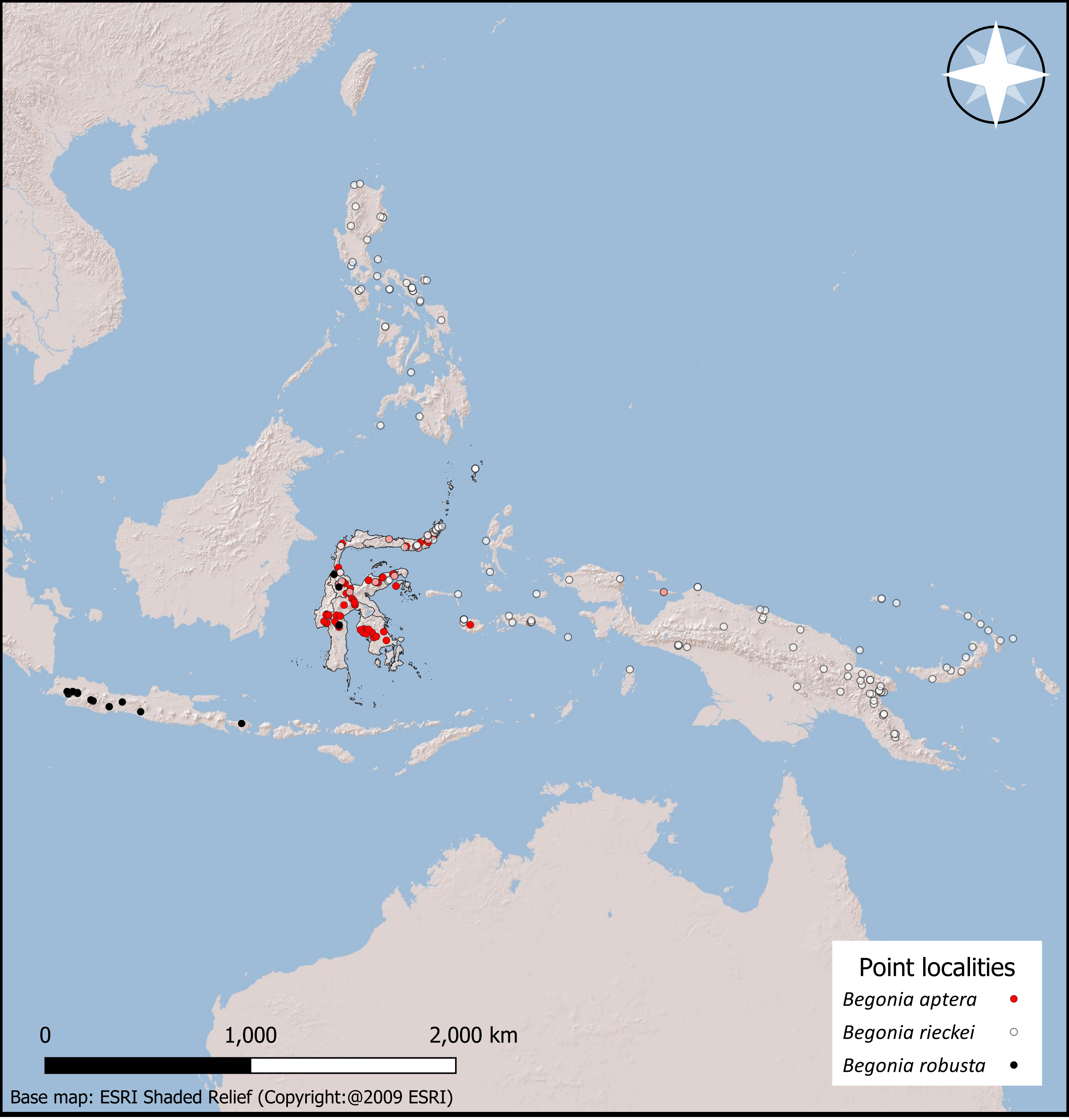


Supplementary Figure 13. Distribution map. Pink colour indicates overlap of occurrences of Begonia aptera (red) and B. rieckei (white).
